# A Mathematical Model of Neonatal Rat Atrial Monolayers with Constitutively Active Acetylcholine-Mediated K^+^ Current

**DOI:** 10.1371/journal.pcbi.1004946

**Published:** 2016-06-22

**Authors:** Rupamanjari Majumder, Wanchana Jangsangthong, Iolanda Feola, Dirk L. Ypey, Daniël A. Pijnappels, Alexander V. Panfilov

**Affiliations:** 1 Laboratory of Experimental Cardiology, Department of Cardiology, Heart Lung Center Leiden, Leiden University Medical Center, Leiden, the Netherlands; 2 Department of Physics and Astronomy, Ghent University, Ghent, Belgium; 3 Moscow Institute of Physics and Technology, (State University), Dolgoprudny, Moscow Region, Russia; King's College London, UNITED KINGDOM

## Abstract

Atrial fibrillation (AF) is the most frequent form of arrhythmia occurring in the industrialized world. Because of its complex nature, each identified form of AF requires specialized treatment. Thus, an in-depth understanding of the bases of these arrhythmias is essential for therapeutic development. A variety of experimental studies aimed at understanding the mechanisms of AF are performed using primary cultures of neonatal rat atrial cardiomyocytes (NRAMs). Previously, we have shown that the distinct advantage of NRAM cultures is that they allow standardized, systematic, robust re-entry induction in the presence of a constitutively-active acetylcholine-mediated K^+^ current (*I*_*KACh-c*_). Experimental studies dedicated to mechanistic explorations of AF, using these cultures, often use computer models for detailed electrophysiological investigations. However, currently, no mathematical model for NRAMs is available. Therefore, in the present study we propose the first model for the action potential (AP) of a NRAM with constitutively-active acetylcholine-mediated K^+^ current (*I*_*KACh-c*_). The descriptions of the ionic currents were based on patch-clamp data obtained from neonatal rats. Our monolayer model closely mimics the action potential duration (APD) restitution and conduction velocity (CV) restitution curves presented in our previous *in vitro* studies. In addition, the model reproduces the experimentally observed dynamics of spiral wave rotation, in the absence and in the presence of drug interventions, and in the presence of localized myofibroblast heterogeneities.

## Author Summary

A fundamentally important element in cardiac *in silico* research is a model for the cardiac cell. It provides a link between measurable characteristics at the subcellular level and biological processes at the whole cell level, thereby allowing the researcher to study mechanisms of cardiac arrhythmias from a molecular cell biological perspective. Such studies are of vast importance for the advancement of understanding of living systems from cells to patient populations. This paper is a joint *in silico*-experimental study in which we propose the first model for the action potential of an NRAM. To develop this model, we fitted patch-clamp data from recent literature, while additionally performing specific measurements of *I*_*KACh-c*_ in NRAMs. *I*_*KACh-c*_ is an important factor in atrial arrhythmogenesis and a promising target for pharmacological AF-management. The model reproduces *in vitro* results such as standard characteristics of AP morphology, restitution, and spiral wave dynamics in monolayers, with effects of a subsequent drug-intervention and in the presence of localized myofibroblast heterogeneities. Thus it can be used as a tool to provide computational support to a variety of systematic experimental studies that investigate the mechanisms underlying atrial fibrillation (AF) in NRAM cultures.

## Introduction

Atrial fibrillation (AF) is the most frequent form of arrhythmia occurring in patients with or without the risk for heart failure [1–8]. Each identified form of AF (paroxysmal, persistent, or chronic) requires specialized treatment. Therefore, an in-depth understanding of the underlying mechanisms is required for optimal management in the treatment of such arrhythmias.

Chronic AF is known to induce functional tissue remodeling through changes in ion channel expression and activity in the cardiomyocyte membranes. Of the various ion channels that have been identified in atrial cardiomyocytes, one family of particular interest is the Kir3.x. These channels produce an inwardly-rectifying K^+^ current (*I*_*KACh*_) that is normally controlled by vagal nerve activity through its transmitter acetylcholine (ACh). There is growing consensus that during chronic AF, *I*_*KACh*_ becomes constitutively active (*I*_*KACh-c*_) [9–11] and hyperpolarizes the cell membrane. This leads to abbreviation of the action potential duration (APD), and stabilization of reentrant electrical circuits that characterize AF [1]. However, despite decades of research on a variety of mammalian hearts, the role of ion-channel remodeling in AF, remains incompletely understood.

Development of a better biophysical understanding of ion channel remodeling in AF requires thorough *in vivo* investigations in the human heart. However, a more practical approach involves studying AF in cell cultures *in vitro*, prior to *ex vivo* and *in vivo* trials. Primary cultures of neonatal rat atrial cardiomyocytes (NRAMs) have been used by researchers interested in cell signaling [11–13]. Recently, we have demonstrated that NRAMs can generate *I*_*KACh-c*_, in primary cultures, thereby making them attractive sources for arrhythmogenic substrates that can be used to study chronic AF [11].

Although *in vitro* and *in vivo* studies have often provided significant insights into the mechanisms underlying reentry initiation in both healthy and diseased cardiac tissue, they have a few limitations. One cannot easily address complex questions related to electrophysiology, that would involve challenging techniques like patch- clamp in parallel with optical mapping measurements, or sub-surface measurements of electrical signals in intact cardiac tissues. Furthermore, independent experimental interventions involving gradual modifications of cell properties, such as gap junctional conductance, coupling between different cells types, localized changes in conductivities of specific ionic channels etc. are very difficult to obtain in an experiment. This is where computer models find wide-scale applicability, from basic science [14–23] to direct clinical applications [24–33].

The basis for any computational study in cardiac tissue is a model for the cardiac cell. However, a model for the NRAM is still lacking. Development of this model is important, because it would allow researchers to perform specific *in silico* studies next to *in vitro* experiments, thereby increasing the possibilities for the development of biological insight into arrhythmias like AF.

In this study, we formulate the first complex ionic model for NRAM monolayers, based on patch-clamp data from recent literature. To remain consistent with *in vitro* experiments, we incorporate natural cellular heterogeneity, and a baseline 15–20% randomly distributed myofibroblasts into these monolayers. We then generate spiral waves in these monolayers by burst pacing (BP) and reproduce spiral wave dynamics as seen in experiments, both under normal conditions and under pharmacological inhibition. In particular, we simulate the effect of the atrial-specific drug tertiapin-Q, a blocker of *I*_*KACh*_, on BP-induced arrhythmias, as reported by Bingen et al. [11] In addition, we reproduce spiral wave dynamics in a monolayer with localized myofibroblast heterogeneity.

## Results

### Formulation of the single cell ionic model

#### General

**Fig 1:**
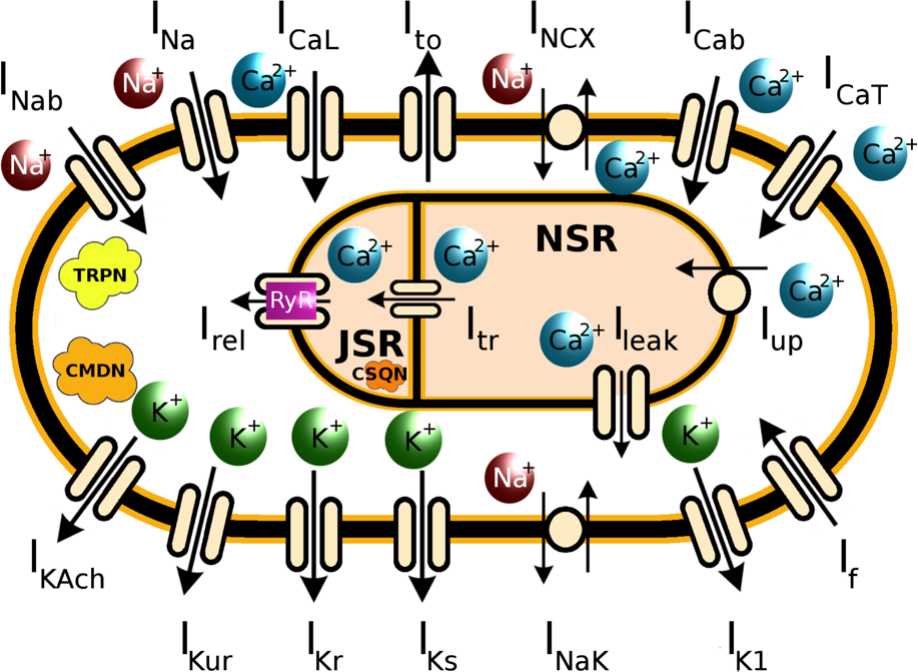
Schematic diagram of the model of a cultured neonatal rat atrial cardiomyocyte (NRAM). The model includes fast Na^+^ current (*I*_*Na*_), background Na^+^ current (*I*_*Nab*_), *L-type* Ca^2+^ current (*I*_*CaL*_), *T-type* Ca^2+^ current (*I*_*CaT*_), transient outward K^+^ current (*I*_*to*_), background Ca^2+^ current (*I*_*Cab*_), hyperpolarization-activated funny current (*I*_*f*_), inward-rectifier K^+^ current (*I*_*K1*_), rapid and slow delayed rectifier K^+^ currents (*I*_*Kr*_ and *I*_*Ks*_), ultra-rapid outward K^+^ current (*I*_*Kur*_), Na^+^/Ca^2+^ exchanger (*I*_*NCX*_*)*, Na^+^/K^+^-ATPase (*I*_*NaK*_), and a constitutively-active acetylcholine-mediated K^+^ current (*I*_*KACh-c*_). The sarcoplasmic reticulum (SR) is divided into 2 compartments for uptake (NSR) and release (JSR). Intracellular Ca^2+^ is transported from the NSR to the JSR via diffusion (*I*_*tr*_). In JSR, the Ca^2+^ is buffered by calsequestrin (CSQN), and released into the cytosol (*I*_*rel*_) via ryanodine receptors (RyR). While most of the intracellular Ca^2+^ is pumped into the NSR via the SERCA (*I*_*up*_), some fluxes passively diffuse back to the cytosol (*I*_*leak*_). The Ca^2+^ in the cytosol is buffered by troponin (TRPN) and calmodulin (CMDN).

The mathematical model of the native NRAMs was developed from an electrical circuit of the cell membrane. It consists of a membrane capacitance, in parallel with voltage-dependent conductance branches that include batteries, and with pumps and exchangers. Thus, the electrophysiological behavior of a single cardiomyocyte can be described using the following ordinary differential equation,

$$\frac{dV}{dt}=\frac{-I_{ion}+I_{stim}}{C_{m}},$$ (1)

$$I_{ion}=I_{Na}+I_{CaL}+{\bar{I}}_{K1}+I_{to}+I_{CaT}+I_{Cab}+I_{NCX}+I_{NaK}+I_{f}+I_{Nab}+I_{Ksus}+I_{KACh},$$ (2)

where *V* is the transmembrane voltage in millivolts (mV), *t* is time in milliseconds (ms), *I*_*ion*_ is the total transmembrane ionic current density in microamperes per square centimeter (μA/cm^2^), *I*_*stim*_ is the externally applied stimulus current, and *C*_*m*_ is the cell capacitance per unit surface area in microfarad per square centimeter (μF/cm^2^). In our simulations with the single cell, we applied a stimulus current of strength 7 pA for a duration of 5 ms. *I*_*Na*_ represents the fast Na^+^ current, *I*_*CaL*_, the *L-type* Ca^2+^ current, *I*_*K1*_, the inward-rectifier K^+^ current, *I*_*to*_, the transient outward *K*^*+*^ current, *I*_*CaT*_, the *T-type* Ca^2+^ current, *I*_*Cab*_, the background Ca^2+^ current, *I*_*NCX*_, the Na^+^/Ca^2+^ exchanger current, *I*_*NaK*_, the Na^+^/K^+^ pump current, *I*_*f*_, the hyperpolarization-activated funny current, *I*_*Nab*_, the background Na^+^ current, and *I*_*KACh*_, the acetylcholine-mediated K^+^ current. All conductances *G*_*X*_ were measured in nanosiemens per picofarad (nS/pF), while intracellular and extracellular ionic concentrations ([*X*]_*i*_, [*X*]_*o*_) were expressed in millimoles per liter (mM). The detailed equations for each of the ionic currents, are specified in the S1 Appendix of the supplementary material, along with a list of model parameters and initial values. The schematic diagram of the cell is shown in Fig 1.

#### Membrane currents

*Fast Na*^*+*^ *current (I*_*Na*_*)*: *I*_*Na*_ was modeled according to the three-gate formulation first used by Beeler and Reuter [34].

$$I_{Na}=G_{Na}m^{3}hj\left(V-E_{Na}\right),$$ (3)

**Fig 2:**
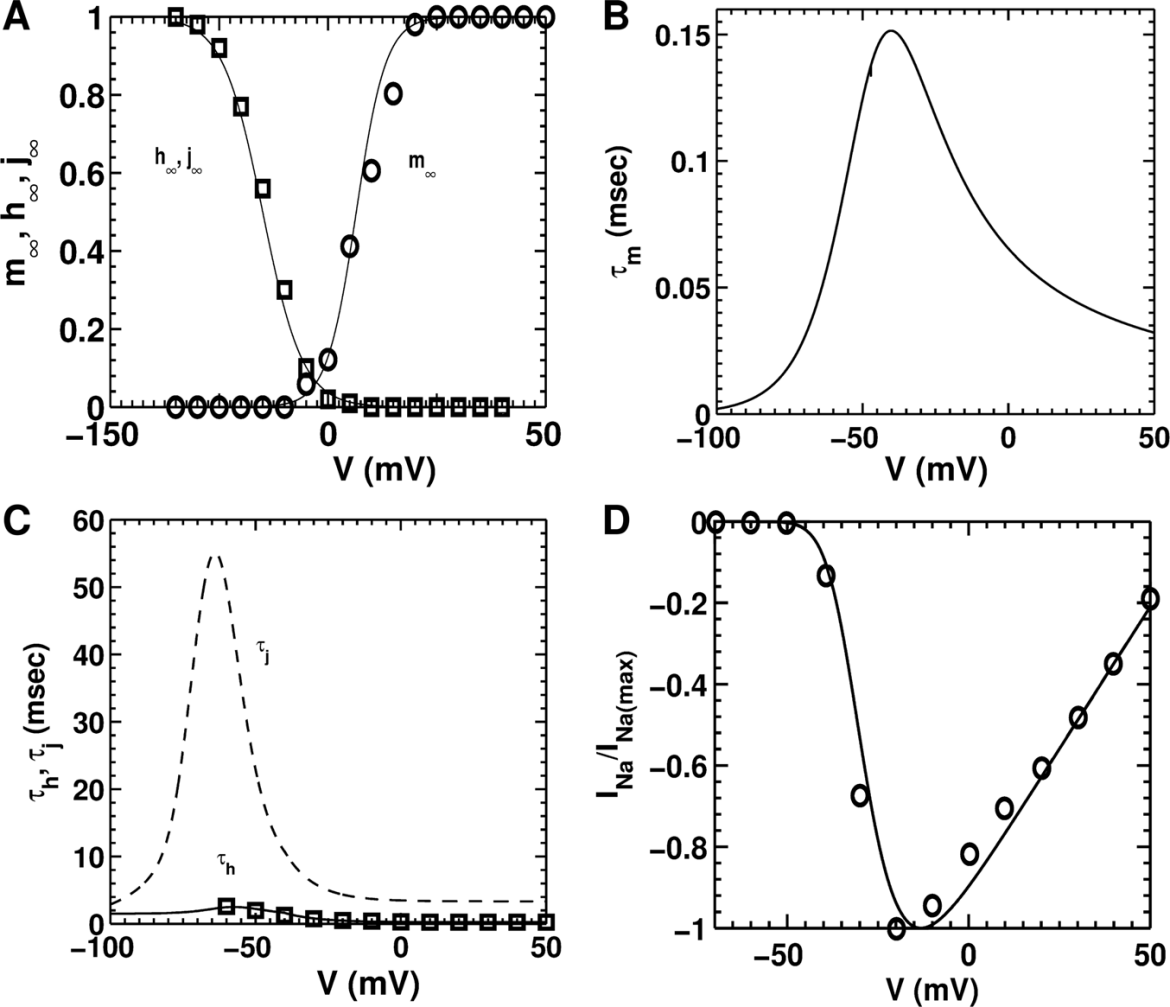
Steady-state characteristics and time constants of fast Na^+^ current (*I*_*Na*_). **A,** comparison of model-generated steady-state activation (m_∞_) curve with experimental data (open circles) from Ramos-Mondragόn et al., [35] and model-generated inactivation (h_∞_, j_∞_) curves with experimental data (open squares) by Voitychuk et al. [36] **B,** activation time constant (*τ*_*m*_) used in the model. **C,** inactivation time constants (*τ*_*h*_ and *τ*_*j*_) from model, with experimental validation using data derived from Voitychuk et al. [36] **D,** normalized *I-V* curve for fast (*I*_*Na*_), overlaid on patch-clamp data derived from Ramos-Mondragόn et al. [35].

Here *m* represents the activation gate, *h*, the fast inactivation gate, and *j*, the slow inactivation gate. The formulation for the channel kinetics was based on experimental data from Ramos-Mondragόn et al., [35] and Voitychuk et al. [36] We used the normalized activation (control) data from Fig 4D of Ramos-Mondragόn et al., [35] to construct the steady-state activation curve (m_∞_) of our model. For steady-state inactivation, we used the (control) data from Fig 5D of Voitychuk et al. [36] Fig 2A shows the steady-state activation and inactivation curves generated using our model, compared with the activation and inactivation kinetics from Ramos-Mondragόn et al. [35] and Voitychuk et al., [36] respectively. The time constants for the activation and slow inactivation gates are taken from the Luo-Rudy guinea-pig ventricular cell model, [37] and scaled appropriately for room temperature [38]. We retain these values for the time constants until more experimental evidence is available (Fig 2B and 2C). The time constant for fast inactivation was modeled on the basis of data derived from Fig 5E of Voitychuk et al., (Fig 2C) [36]. The maximal conductance (*G*_*Na*_) was determined by fitting the maximum upstroke velocity (dV/dt_max_) that we measured *in vitro*. This value was found to be in consonance with the recordings of Voitychuk et al. [36] Finally, current-voltage relationships (*I-V* curves) were constructed from the model formulation and fitted with experimental data from Fig 4B of Ramos-Mondragόn et al., (Fig 2D) [35]. The individual equations for these three gates governing Na^+^-channel kinetics, together with the time constants and initial values, are provided in the S1 Appendix of the supplementary material.

*L-type Ca*^*2+*^ *current (I*_*CaL*_*)*: Although *I*_*CaL*_ plays an important role in the generation of an AP in ventricular cells, in atrial cells its magnitude is comparatively less (*cf*. Figs 2B and 3B of Avila et al., [39]). Furthermore, the electrophysiological influence of L-type Ca^2+^ in NRAMs is not well studied. Therefore, we used a more general formulation of *I*_*CaL*_, namely, the one according to the model by ten Tusscher et al. [40]

$$I_{CaL}=4G_{CaL}dff_{Ca}\frac{VF^{2}}{RT}\frac{\left({\left[Ca\right]}_{i}e^{\frac{2VF}{RT}}-0.341{\left[Ca\right]}_{o}\right)}{e^{\frac{2VF}{RT}}-1},$$ (4)

**Fig 3:**
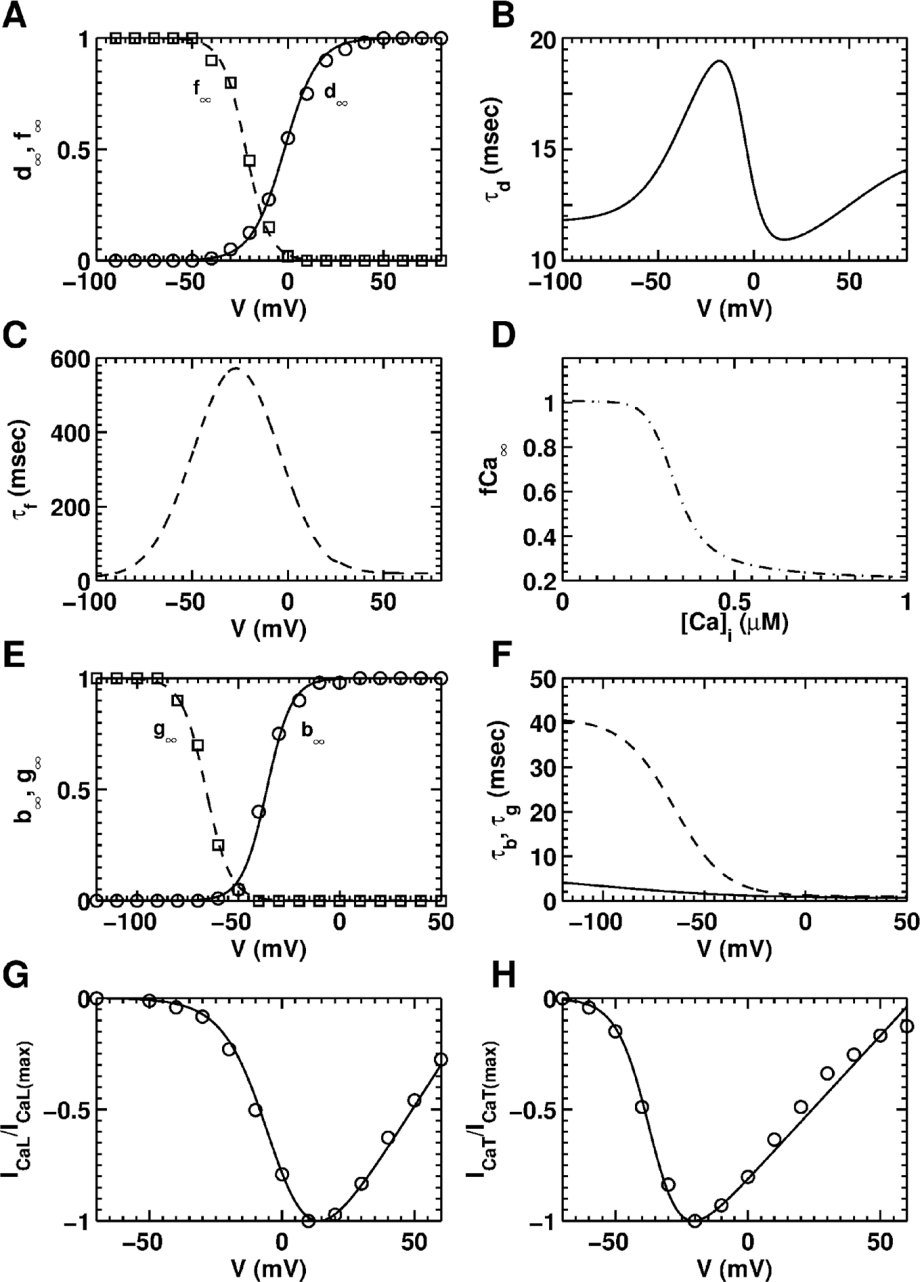
Steady-state characteristics and time constants of the *L-type* and *T-type* Ca^2+^ currents (*I*_*CaL*_ and *I*_*CaT*_, respectively). **A,** steady-state activation (*d*_*∞*_) and voltage-dependent inactivation (*f*_*∞*_) curves of *I*_*CaL*_. **B,** activation time constant (*τ*_*d*_) of *I*_*CaL*_. **C,** inactivation time constant (*τ*_*f*_) of *I*_*CaL*_. **D,** Ca^2+^-dependent steady-state inactivation (*f*_*Ca∞*_) curve of *I*_*CaL*_. **E,** steady-state activation (*b*_*∞*_) and inactivation (*g*_*∞*_) curves of *I*_*CaT*_. **F,** activation (*τ*_*b*_) and inactivation (*τ*_*g*_) time constants of *I*_*CaT*_. **G,** normalized *I-V* curve for *I*_*CaL*_ and (**H)** normalized *I-V* curve for *I*_*CaT*_. Black lines represent data generated using the model, whereas open circles and squares represent data derived from patch-clamp experiments of Avila et al. [39]

It consists of a voltage-dependent activation gate *d*, a voltage-dependent inactivation gate *f*, and an intracellular calcium-dependent inactivation gate *f*_*Ca*_. The driving force was modeled with a Goldmann-Hodgkin-Katz equation, ignoring the permeability for the Na^+^ and K^+^ ions. The steady-state activation and inactivation curves for the voltage-dependent gates (i.e., *d*_*∞*_ and *f*_*∞*_) were obtained by fitting the patch-clamp (control) data from the experiments of Avila et al, on NRAMs (Fig 3A) [39]. Unfortunately, no literature was available for the activation and inactivation times of *I*_*CaL*_ in NRAMs. Thus in our model, we retained the activation and inactivation time constants from the neonatal rat ventricular cardiomyocyte (NRVM) model by Korhonen et al. [41] (Fig 3B and 3C), with a 10 ms correction to the activation time constant (*τ*_*d*_). Korhonen et al. [41] obtained the value for Ca^2+^-independent inactivation from the experimental records of Pignier et al., [42] who used NRVMs for their measurements. The formulation for the [*Ca*]_*i*_-dependent inactivation was also taken from the NRVM model by Korhonen et al., [41] (Fig 3D). The maximal conductance for the *L-type* Ca^2+^ channel was adjusted along with maximal conductances of the *I*_*K1*_, till a reasonable fit of the control APD_80_ restitution curve was obtained. The normalized *I-V* curve for *I*_*CaL*_, as obtained from the present model is shown in Fig 3G and overlaid on experimental data derived from Avila et al. [39]

*T-type Ca*^*2+*^ *current (I*_*CaT*_*)*: The T-type Ca^2+^ current was formulated on the basis of Dokos et al., [43] as:

$$I_{CaT}=G_{CaT}bg\left(V-E_{Ca}+106.5\right),$$ (5)

where *b* represents the voltage-dependent activation gate and *g*, the voltage-dependent inactivation gate. The steady-state activation and inactivation curves were obtained by fitting the (control) data from the experiments of Avila et al., (Fig 3E) [39]. As in the case of *I*_*CaL*_, data on activation and inactivation times of *T-type* Ca^2+^ channels are still lacking. Therefore, we used time constants (τ_b_ and τ_g_) from the NRVM model by Korhonen et al. (Fig 3F) [41] The maximal conductance (*G*_*CaT*_) was adopted from the NRAM data of Avila et al. [39] The normalized *I-V* curve for *I*_*CaT*_ generated using our model is shown in Fig 3H, together with *I-V* curve data derived from control experiments of Avila et al., on NRAMs [39].

**Fig 4:**
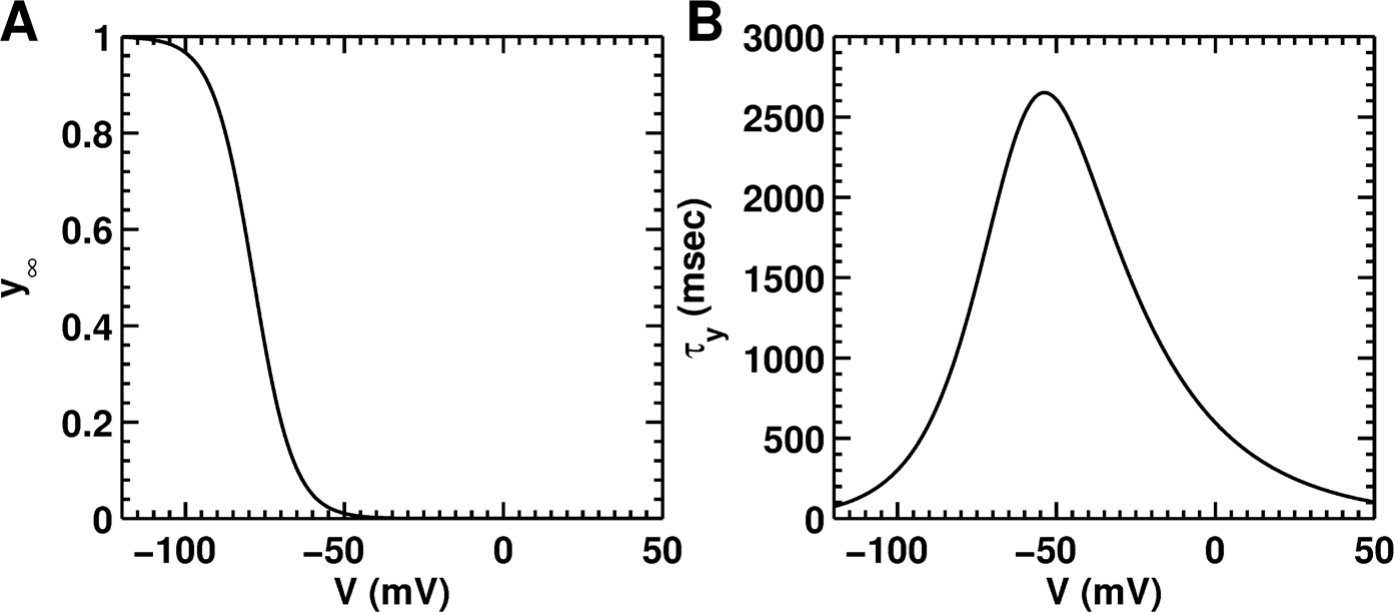
Kinetics of hyperpolarization-activated funny current (*I*_*f*_*)* generated using the model. **A,** steady-state activation curve (*y*_*∞*_). **B,** activation time constant (*τ*_*y*_), as a function of *V*.

*Background currents (I*_*Cab*_, *and I*_*Nab*_*)*, *and hyperpolarization-activated funny current (I*_*f*_*)*: Two background currents were directly taken from the adult rat ventricular myocyte model by Pandit et al. [44] These are linear (Ohmic) currents, representing the leakage of ions through non-specific ion channels in the NRAM-membrane. The maximal conductances of these are chosen such as to obtain the correct resting membrane potential, and are listed in the S1 Appendix of the supplementary material. There is substantial evidence regarding the presence of a hyperpolarization-activated, funny current (*I*_*f*_) in NRVMs [45–48]. However, whether this current is also present in NRAMs is unclear. In line with the concept that it is present in both sino-atrial nodal cells and ventricular cells, we assume that it is also present in the atrial cells, but perhaps, not so significantly. Therefore, we include *I*_*f*_ in the NRAM model, based on the formulation from the ventricular model by Korhonen et al. [41] The activation kinetic of the voltage-dependent channel *y* is shown in Fig 4A, together with the voltage-dependency of its time constant *τ*_*y*_ (Fig 4B). We adopted the same value of the maximal ionic conductance for *I*_*f*_ as Korhonen et al. [41] However, the presence or absence of this current did not make any significant difference to the morphology and characteristics of the AP or to the resting membrane potential.

*Transient outward K*^*+*^ *current (I*_*to*_*)*: The transient outward K^+^ current was modeled as:

$$I_{to}=G_{to}r\left(0.706s+0.294s_{slow}\right)\left(V-E_{K}\right),$$ (6)

**Fig 5:**
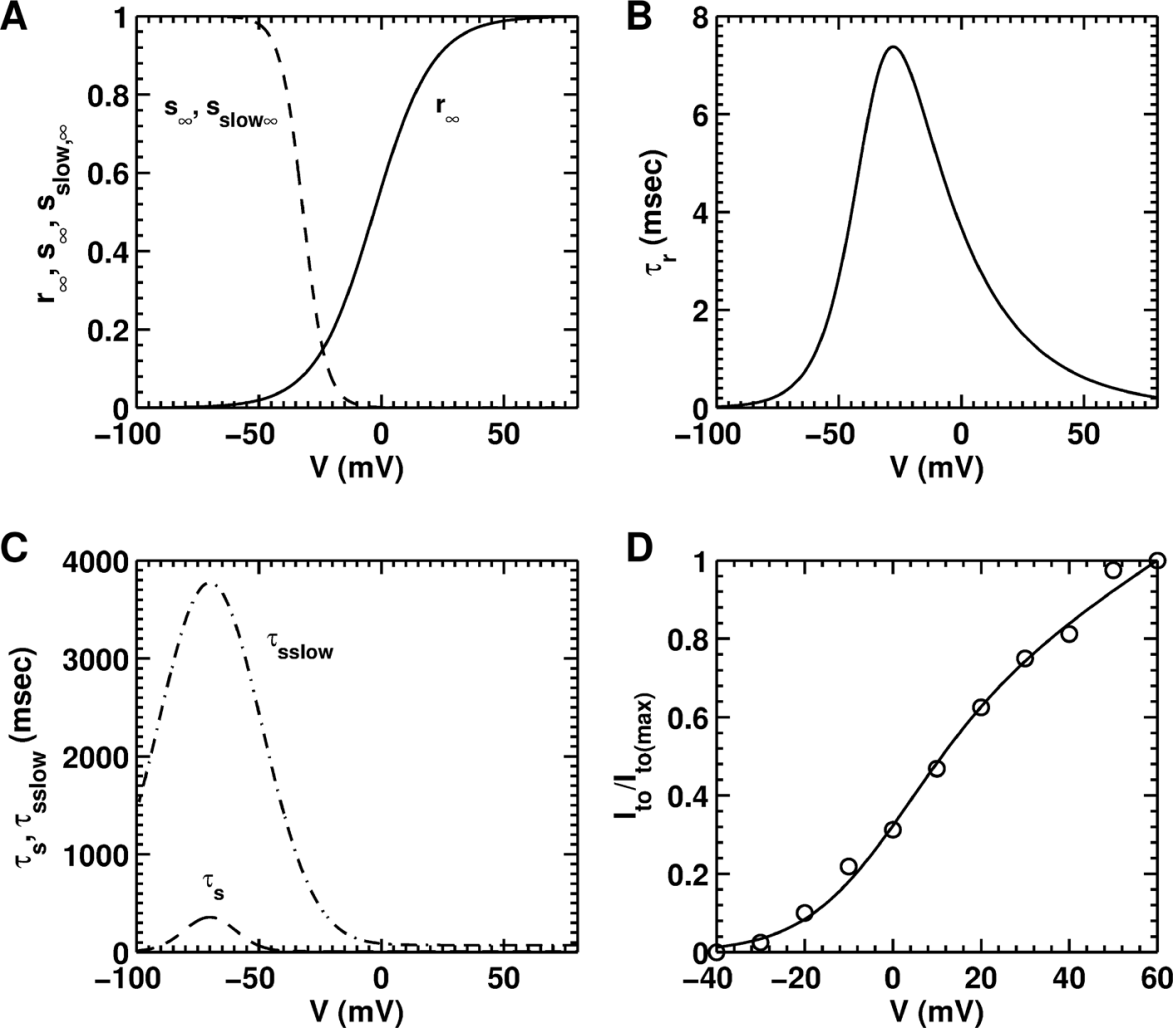
Steady-state characteristics and time constants of the transient outward K^+^ current (*I*_*to*_) generated using the model. **A,** steady-state activation (*r*_*∞*_) and fast and slow inactivation (*s*_*∞*_, *s*_*slow∞*_) curves. **B,** activation time constant of the model. **C,** inactivation time constants (*τ*) from model. **D,** normalized peak *I-V* curve for (*I*_*to*_), overlaid on patch-clamp data (open circles) derived from Ramos-Mondragόn et al. [35]

It comprises a voltage-dependent activation gate *r*, a voltage-dependent fast inactivation gate *s*, and a voltage-dependent slow inactivation gate *s*_*slow*_. A comparison of *I-V* curves for outward K^+^ currents recorded from NRAMs and NRVMs show that the trends in *I*_*to*_ are the same in both cell-types (*cf*. Fig 4B of Saygili et al., [49] and Fig 1B of Cahill et al. [48]), although the magnitudes are different (ventricular cells produce more *I*_*to*_ than atrial cells). Thus, we used the same formalism for the steady-state activation (*r*_*∞*_) and inactivation curves (*s*_*∞*_ and *s*_*slow∞*_), and the activation (*τ*_*r*_) and inactivation time constants (*τ*_*s*_, and *τ*_*slow*_), as in the rat ventricular cardiomyocyte model by Pandit et al. [44] The ion channel kinetics for the transient outward K^+^ current are shown in Fig 5A–5C. Fig 5D shows the normalized *I-V* curve for simulated *I*_*to*_, overlaid on experimental data derived from Ramos-Mondragόn et al. [35]

*Sustained outward K*^*+*^ *current (I*_*Ksus*_*)*: In order to compare the *I-V* curve generated by the model, with *I-V* curve data derived from the control experiments of Ramos-Mondragόn et al., on NRAMs [35], the sustained outward K^+^ current was modeled as a sum of three currents: the ultra-rapid current *I*_*Kur*_, the rapid-delayed rectifier current *I*_*Kr*_, and the slow-delayed rectifier current *I*_*Ks*_.

$$I_{Ksus}=0.16\left(I_{Kur}+I_{Kr}+I_{Ks}\right)$$ (7)

**Fig 6:**
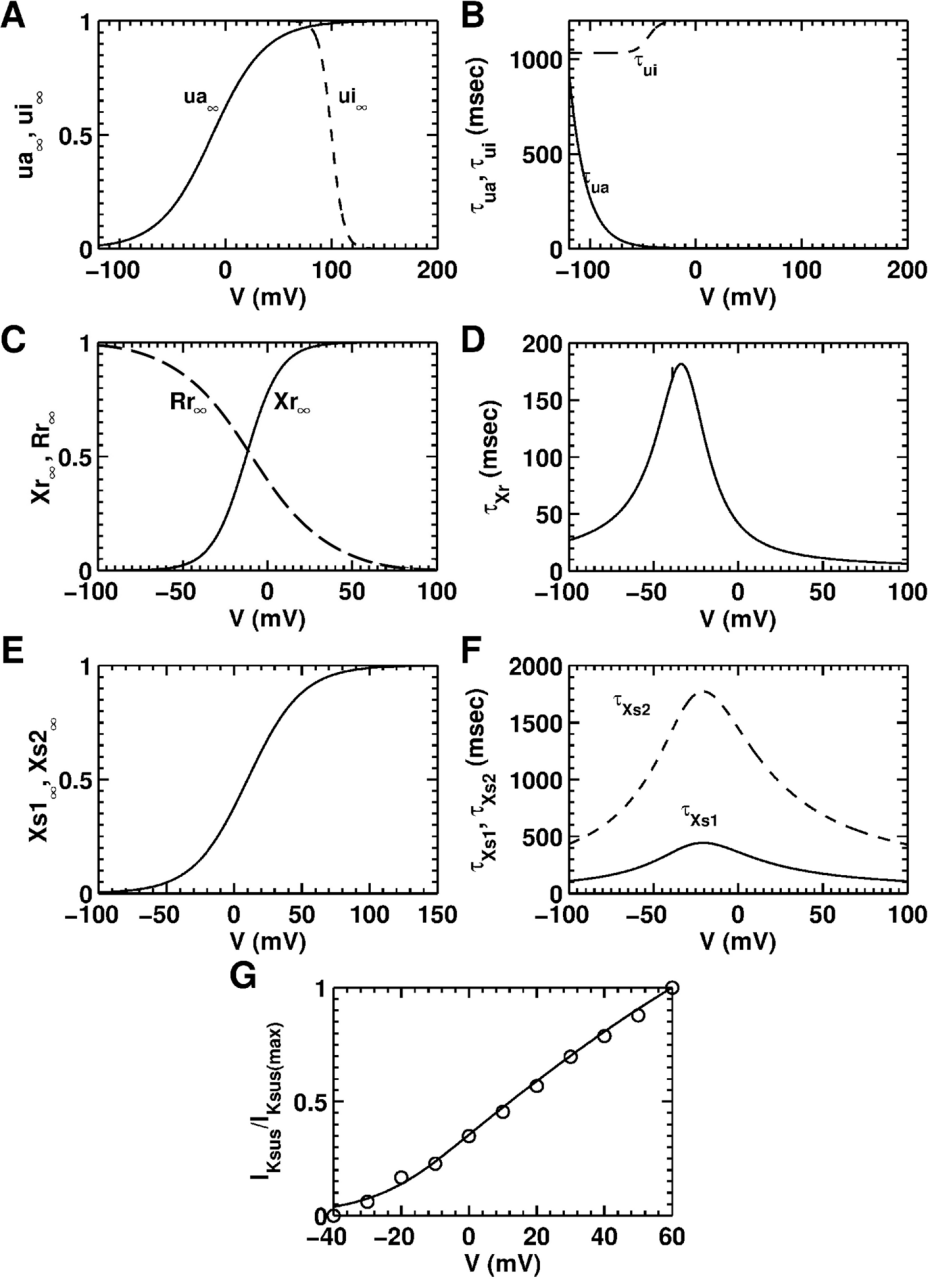
Steady-state characteristics and time constants of the sustained K^+^ current (*I*_*Ksus*_) generated using the model. The sustained current was a composition of the ultra-rapid K^+^ current (*I*_*Kur*_), the rapid-delayed rectifier K^+^ current (*I*_*Kr*_), and the slow-delayed rectifier K^+^ current (*I*_*Ks*_). **A,** steady-state activation (*ua*_*∞*_) and inactivation (*ui*_*∞*_) curves of *I*_*Kur*_. **B,** activation (*τ*_*ua*_) and inactivation (*τ*_*ui*_) time constants of *I*_*Kur*_. **C,** steady-state activation (*Xr*_*∞*_)and inactivation (*Rr*_*∞*_) curves of *I*_*Kr*_. **D,** activation time constant (*τ*_*Xr*_) of *I*_*Kr*_. **E,** steady-state activation curves (*Xs1*_*∞*_ and *Xs2*_*∞*_) of *I*_*Ks*_. **F,** fast (*τ*_*Xs1*_) and slow (*τ*_*Xs2*_) inactivation time constants of *I*_*Ks*_. **G,** normalized *I-V* curve for sustained K^+^ current (*I*_*Kur*_ *+ I*_*Kr*_ *+ I*_*Ks*_), overlaid on patch-clamp data (open circles) derived from Ramos-Mondragόn et al. [35]

The formulation for the first component *I*_*Kur*_ was taken from the Bondarenko model [50]. The second and third components of *I*_*Ksus*_, namely *I*_*Kr*_ and *I*_*Ks*_, were taken from the NRVM model by Hou et al. [51] All three currents are described in the S1 Appendix of the supplementary material. The maximal conductances for the three components (*G*_*Kur*_, *G*_*Kr*_, and *G*_*ks*_) were adopted directly from the ventricular model by Hou et al. [51] However, the net sustained current *I*_*Ksus*_ was adjusted (by the factor 0.16 in Eq 7) to reproduce the measured control APD_80_ restitution curve. Steady-state activation and inactivation kinetics of the ionic conductances involved in producing the three component currents are illustrated in Fig 6A–6F. Fig 6G shows the normalized *I-V* characteristic curve for simulated *I*_*Ksus*_, overlaid on experimental data derived from Ramos-Mondragόn et al. [35]

*Inward-rectifier current and K*^*+*^ *leak (*${\bar{I}}_{K1}$*)*: The NRAMs showed less inward-rectifier current than the NRVMs. For *I*_*K1*_, the basic formulation of the model by Hou et al. [51] was used, with additional adjustments based on our patch-clamp recordings:

$${\bar{I}}_{K1}=0.048925\left(\frac{K_{o}}{K_{o}+210}\right)\left(\frac{V-E_{K}-10}{1.0+exp^{0.041\left(V-E_{K}-10\right)}}\right)+0.01\left(V-E_{K}\right),$$ (8)

**Fig 7:**
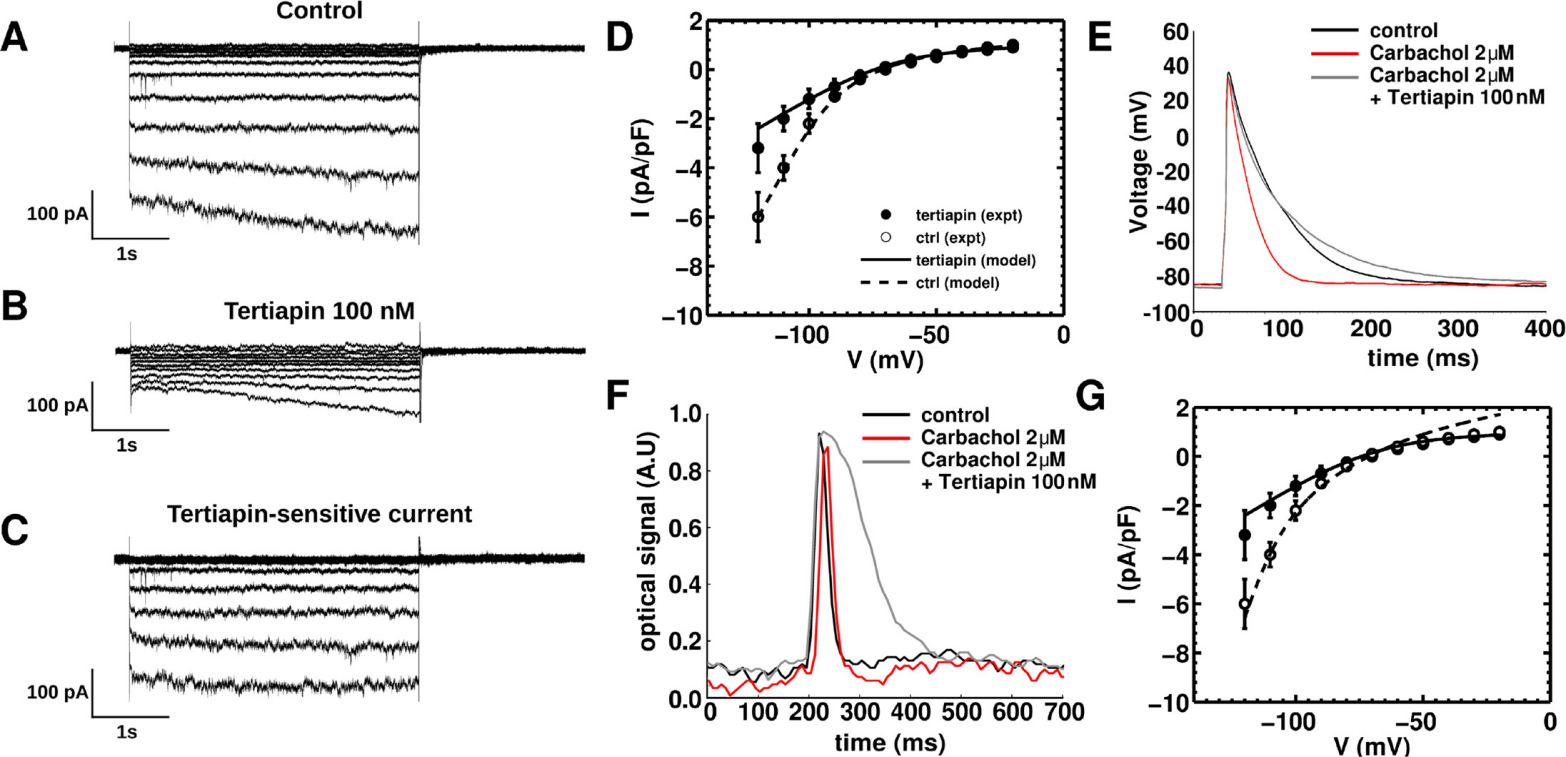
The time-dependent hyperpolarization-activated current (*I*_*KH*_) and its block by tertiapin. **A,** The voltage-clamp recordings depict large inward currents evoked by hyperpolarizations (5s), whereas, very little current was activated during steps to -50 mV and more positive potentials. In addition to pharmacological blocking, while performing voltage-clamp recording, Na^+^ current (*I*_*Na*_) and T-type Ca^2+^ current (*I*_*CaT*_) were inactivated by holding the cells at the potential of -40 mV. **B,** In the presence of tertiapin (100 nM), which selectively suppresses the constitutively active acetylcholine (ACh)-mediated K^+^ current *I*_*KACh-c*_ component of *I*_*KH*_, only inward rectifier current (*I*_*K1*_) is left with K^+^ leak. **C,** Tertiapin-sensitive currents or *I*_*KACh-c*_ (**B** subtracted from **A**). **D,** comparison of *I-V* characteristics, generated by our model (dashed line for control and solid line for tertiapin-Q treatment) and mean±SEM (open circles for control and filled circles for tertiapin-Q treatment) current density based on 8 neonatal rat atrial cardiomyocytes (NRAMs). **E**, representative AP-recordings from a single NRAM in control condition (black), upon treatment with 2 μM Carbachol (red), and upon subsequent treatment with 100 nM tertiapin-Q (gray). **F**, representative AP optical mapping traces recorded from a NRAM monolayer, under control condition (black), upon treatment with 2 μM Carbachol (red) and upon treatment with 100 nM tertiapin-Q (gray). **G**, compensated I-V characteristics for monolayer model, overlaid on the experimental single-cell *I-V* curves of **D**.

The current consists of a [K]_o_-dependent part, a voltage-dependent inward rectification factor, and a linear leakage (last term). Our patch-clamp recordings of the inward-rectifier current were contaminated with a K^+^ leakage current, which shifted the reversal potential of the net measured current by ~10 mV. This shift was also observed in the records of Saygili et al. who showed the complete *I-V* curve for the inward rectifier (*I*_*K1*_) in the NRAMs [49]. Thus, based on our patch-clamp data, the maximal conductance was adjusted to 47.5% of that of a NRVM [51], and the reversal potential for *I*_*K1*_ was shifted by +10 mV, ensuring that the total current ${\bar{I}}_{K1}$ was not a pure inward-rectifier, but a composite of the pure *I*_*K1*_ and the K^+^ leak. An example of the inward rectifier current is shown in Fig 7B.

*Acetylcholine-mediated K*^*+*^ *current (I*_*KACh*_*)*: Unlike the situation in NRVMs, in NRAMs the acetylcholine (ACh)-mediated K^+^ current (*I*_*KACh*_) contributes to the repolarization reserve. We performed whole-cell voltage-clamp experiments, specific for the measurement of this current. We measured *I*_*KACh*_ as the instantaneous time-dependent increase in current upon hyperpolarization *(I*_*KH*_) [52–53]. Hyperpolarizing voltage clamp steps (5s) from -40 mV (to inactivate Na^+^ current and T-type Ca^2+^ current) evoked inward currents. As expected, the current exhibited pronounced inward rectification, with tiny current at potential positive to -50 mV (Fig 7A). The addition of tertiapin-Q (100 nM) to the modified extracellular solution attenuated *I*_*KH*_ (Fig 7B). Using our patch-clamp recordings of the time-dependent hyperpolarization-activated current *I*_*KH*_ in Fig 7A and the inward-rectifier current *I*_*K1*_ in Fig 7B, we obtained the tertiapin-sensitive *I*_*KACh*_ (Fig 7C) by digitally subtracting the currents in Fig 7B from those in Fig 7A. Based on the formulation of Kneller et al. [54] for *I*_*KACh*_ in canine ventricular cardiomyocytes, we fitted the experimental data presented in Fig 7D to obtain an I-V curve for the *I*_*KACh*_ in the present model (Eq 9) with [*ACh*] = 1 μM.

$$I_{KACh}=\left(\frac{3.5}{1.0+\frac{9.13652}{{\left[ACh\right]}^{0.477811}}}\right)\left(0.04+\frac{0.23}{1.0+e^{\left(\frac{V+102}{10}\right)}}\right)\left(V-E_{K}-10\right),$$ (9)

Near-overlap of the *I-V* curves in the physiological range of voltages indicates that in single cells *I*_*KACh*_ is present, but not prominently active (Fig 7D). To verify this finding we performed additional current-clamp experiments. In particular, we evoked APs from 4 different NRAMs, in 4 independent control experiments, followed by subsequent treatment with 2 μM carbachol (the nonselective muscarinic receptor agonist). With this concentration of carbachol, *I*_*KACh*_ is expected to be fully activated, thereby leading to abbreviation of APs in NRAMs. AP recordings, during carbachol-treatment, showed an average ~35% decrease in APD_50_ and an approximate ~40% decrease in APD_80_, confirming that *I*_*KACh*_ was being abundantly activated in NRAMs by carbachol (Fig 7E, red line). Further treatment of these cell with 100 nM tertiapin-Q caused the original AP characteristics to be restored and lengthened of APD_80_ (Fig 7E, gray line). This finding suggests that isolated NRAMs expressed potentially active Kir3.x channels, which contribute to *I*_*KACh*_. However, this current is dormant on single NRAMs to produce substantial amounts of *I*_*KACh-c*_.

In monolayers, however, *I*_*KACh*_ is found to be constitutively active and has been established to play a key role in the development of a proarrhythmic tissue substrate, because block of *I*_*KACh*_ drastically increases APD, whereas further opening with 2 μM carbachol does not have any substantial effect (Fig 7F) [9–10,55]. Previously, we showed that complete block of the Kir3.x channels could lead to drastic changes in the overall electrophysiological behavior of the NRAM monolayer, such as near-tripling of APD_80_, reduction in APD_80_ dispersion, and consequent termination of burst pacing-induced arrhythmias [11]. Therefore, we modified our formulation for the *I*_*KACh*_ in higher dimensional models to make it constitutively active (*I*_*KACh-c*_; Fig 7G). However, measurement of *I*_*KACh*_ at physiological voltages in the presence of Carbachol requires an extracellular environment that contains abnormally high concentrations of K^+^, which would cause us to study a condition that will not reflect the actual situation in monolayers, which we aim to model and study *in silico*. Therefore we modified the single-cell formulation of this current, based on the concept derived from Dobrev et al (see Fig 1A of Dobrev et al.) [9], and increased the gap between the *I-V* curves for *I*_*K1*_ and *I*_*KACh*_ in the physiological range of voltages. The modified formulation is shown in Eq 10.

$$I_{KACh-c}=0.37488\left(0.075+\frac{0.35}{1+e^{\left(\frac{V+102}{10}\right)}}\right)\left(V-E_{K}-10\right),$$ (10)

*Na*^*+*^*/Ca*^*2+*^ *exchanger (I*_*NCX*_*)*, *and Na*^*+*^*/K*^*+*^ *pump (I*_*NaK*_*) currents*: The Na^+^/Ca^2+^ exchanger current was taken from the adult rat ventricular cardiomyocyte model by Pandit et al., [44] whereas the formulation for the Na^+^/K^+^ pump current was adopted from the Luo-Rudy model of guinea pig ventricular cardiomyocyte [37], keeping the same Na^+^ and K^+^ concentration levels as used in our patch-clamp experiments (see Methods section). The formulations for these currents and their maximal conductances are listed in the S1 Appendix of the supplementary material.

#### Intracellular ion dynamics

For simplicity, the intracellular Na^+^ and K^+^ concentrations were kept constant in time. Dynamics of Ca^2+^ changes was adapted from the NRVM model by Korhonen et al. [41] The sarcoplasmic reticulum (*SR*) was modeled as a composition of two compartments, the *SR*_*uptake*_ or the network sarcoplasmic reticulum (*NSR*), and the *SR*_*release*_ or the junctional sarcoplasmic reticulum (*JSR*) (Fig 1). The volume of *JSR* was assumed to be 10% of the total volume of the *SR*. [41] The Ca^2+^ flux between the *NSR* and the cytosol through the SERCA, was adopted from the rabbit ventricular cardiomyocyte model by Shannon et al., [56] and modified in accordance with the NRVM model by Korhonen et al. [41] With the same assumption as Korhonen et al., [41] that the calsequestrin buffer was located close to the ryanodine receptors (RyRs), the complex intermolecular coupling within an RyR cluster was replaced by a single average RyR, and the opening of the RyR was modeled as being regulated by the cytosolic Ca^2+^ concentration near the *SR* and the *JSR*. Our model accounted for Ca^2+^ buffering through troponin and calmodulin.

The origins of the formulations of all the currents and the data used to adjust the model are summarized in Table 1.

**Table 1:** Origins of the formulations of the various ionic currents.

Current(pA/pF)	Formulation			Parameter adjusted	Data used for fitting		
	Reference	Species	cell-type		Reference	Species	Atrial/Ventricular
*I*_*Na*_	Beeler and Reuter. [34]	Human	Purkinje fiber	*m*	Ramos-Mondragόn et al. [35]		
				*h*	Voitychuk et al. [36]	neonatal rat	Atrial
*I*_*CaL*_	ten Tusscher et al. [15]	Human	Ventricular cardiomyocyte	*d*,*f*	Avila et al. [39]	neonatal rat	Atrial
${\bar{I}}_{K1}$	Hou et al. [51]	neonatal rat	Ventricular cardiomyocyte	${\bar{I}}_{K1}$	our experimental data	neonatal rat	Atrial
*I*_*to*_	Pandit et al. [44]	adult rat	Ventricular cardiomyocyte	*G*_*to*_	Ramos-Mondragόn et al. [35]	neonatal rat	Atrial
*I*_*CaT*_	Dokos et al. [43]	rabbit	Sinoatrial node	*b*,*g*	Avila et al. [39]	neonatal rat	Atrial
*I*_*Nab*_ *I*_*Cab*_	Pandit et al. [44]	adult rat	Ventricular cardiomyocyte	*-*	-	-	-
*I*_*NCX*_ *I*_*NaK*_	Luo-Rudy model [37]	guinea pig	Ventricular cardiomyocyte	[*Na*]_o_, [*K*]_*o*_	our patch-clamp experiments	neonatal rat	Atrial
*I*_*f*_	Korhonen et al. [41]	neonatal rat	Ventricular cardiomyocyte	*-*	-	-	-
*I*_*Ksus*_	Bondarenko model. [50] (for *I*_*kur*_)	mouse	Ventricular cardiomyocyte	*G*_*Kur*_	Ramos-Mondragόn et al. [35]	neonatal rat	Atrial
	Hou et al. [51] (for *I*_*Kr*_ and *I*_*Ks*_)	neonatal rat	Ventricular cardiomyocyte	*G*_*Kr*_, *G*_*Ks*_			
*I*_*KACh*_	Kneller et al. [54]	dog	Atrial cells	*I*_*KACh*_	our experimental data	neonatal rat	Atrial

### Features of the single-cell ionic model

#### Single cell

**Fig 8:**
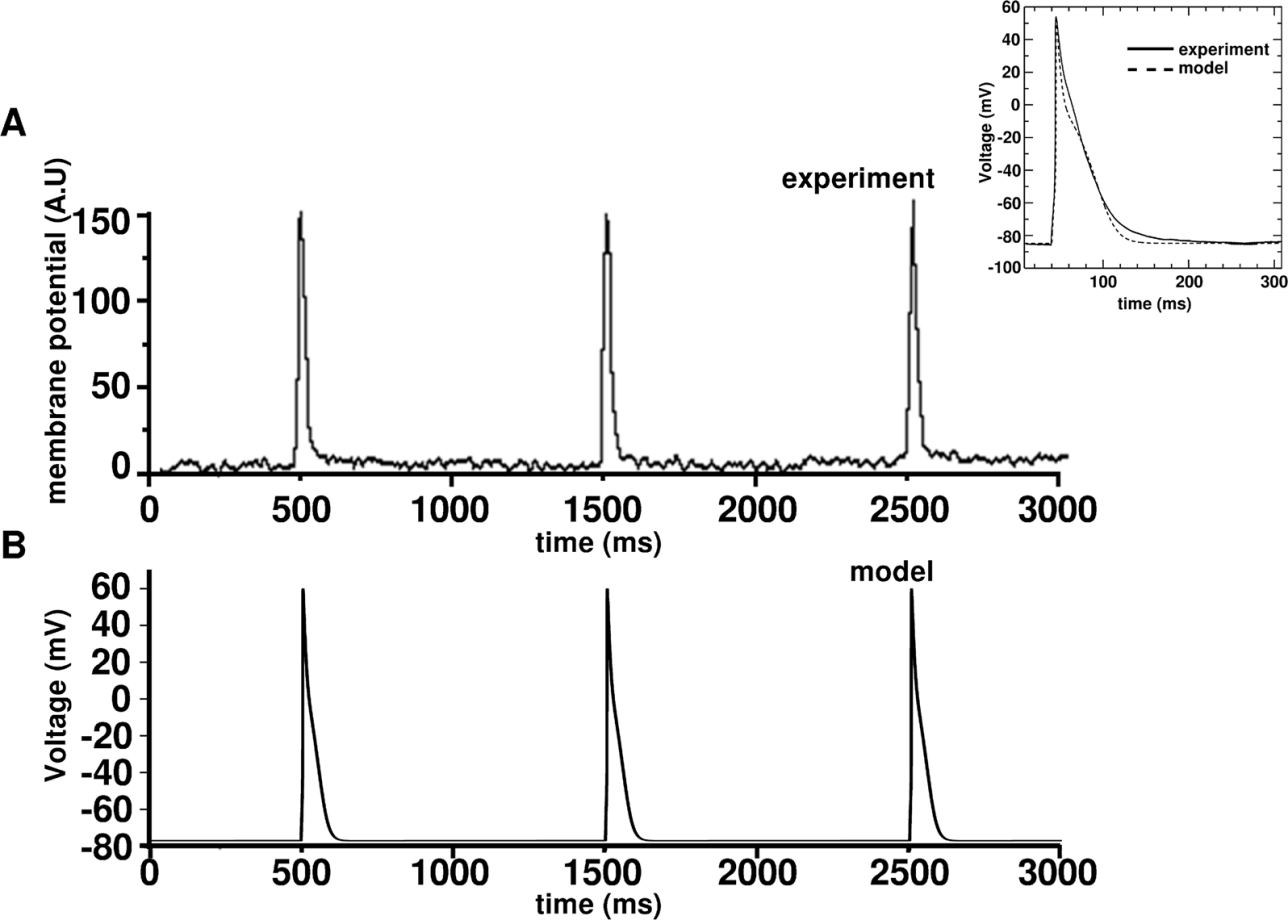
Comparison of model-generated APs with experimental data. **A,** AP optical mapping traces obtained from 1 Hz electrical pacing of a NRAM monolayer **B**, Sequence of APs generated using our monolayer model, also at 1 Hz electrical stimulation. Inset shows steady-state AP generated using our model, overlaid on a representative AP recorded from a NRAM using whole-cell patch-clamp technique. In patch-clamp experiment, the AP was evoked by a single electrical square pulse of strength 100 pA and duration 5 ms, from an artificial resting membrane potential of -85mV, established by means of a holding current.

**Fig 9:**
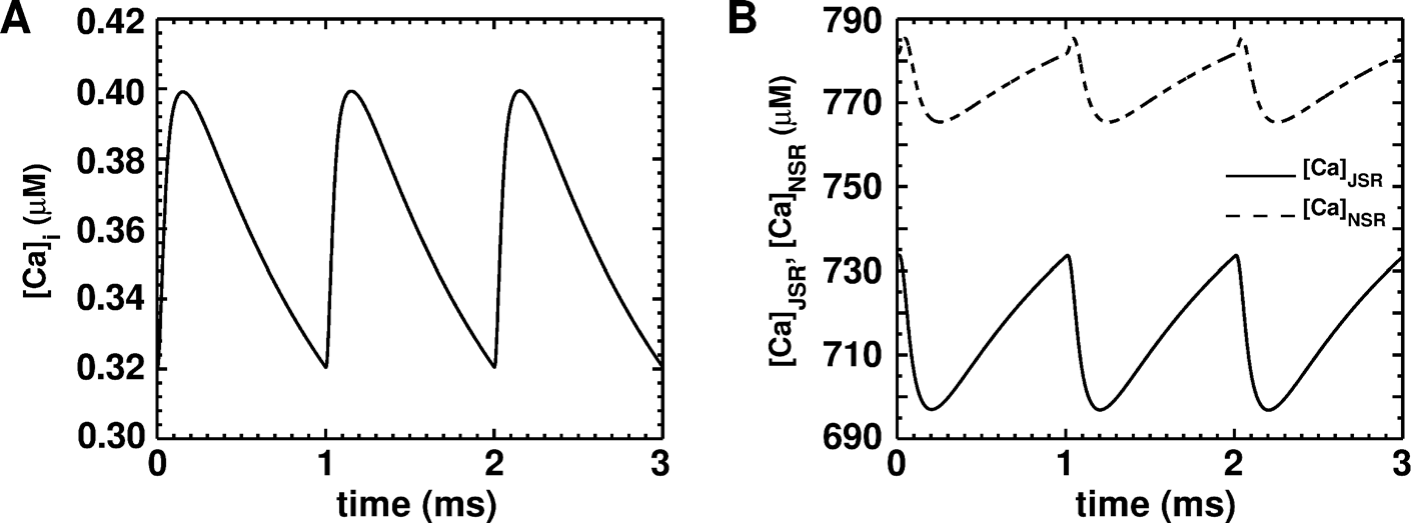
Dynamics of calcium transients evoked by an AP. **A,** time-course of the intracellular free Ca^2+^ concentration ([*Ca*]_*i*_). **B,** time-course of the Ca^2+^ concentrations in the junctional sarcoplasmic reticulum ([*Ca*]_*JSR*_) shown in solid line and the network sarcoplasmic reticulum ([*Ca*]_*NSR*_) shown in dash line.

Fig 8A inset shows an action potential (AP) generated using our model (dashed line), overlaid on an AP measured with whole-cell patch-clamp (solid line), at a membrane potential of ~-85mV, which was established by a holding current to obtain a standard condition for the measurement of excitability. The AP shows the characteristic sharp upstroke, followed by a decay (repolarization phase), with complete absence of a notch and a dome. In the absence of a holding current, the model generated a resting membrane potential (RMP) of -72.0 mV, which was in consonance with the value -73.0 ± 2.5 mV, derived from the patch-clamp measurements of Voitychuk et al. [36] dV/dt_max_ was estimated to be 114 mV/ms, in line with 114.80±24.77 mV/ms measured in our experiments, and those of Voitychuk et al. [36] APD_80_ and APD_50_ also showed good agreement with our experimental values (see Table 2). The calcium transients generated by the model are shown in Fig 9A and 9B. The mean concentrations of Ca^2+^ in the uptake and release compartments were ~775 μM and ~715 μM, respectively, as opposed to their corresponding counterparts of ~750 μM and ~650 μM in ventricular cells [35], The mean free cytoplasmic Ca^2+^ was found to be ~0.36 μM in simulated atrial cells, as opposed to 0.475 μM in ventricular cells (Fig 8A of Korhonen et al. [41]). In general the amplitude of Ca^2+^ cycles was found to be smaller than in neonatal rat ventricular cells [41].

**Table 2:** Comparison of model-generated single-cell AP characteristics with those measured from experiment.

Parameter	Model	Experiment
V_rest_ (mV)	-72.0	-73.0 ± 2.5 (Voitychuk et al. [34])
AP amplitude (mV)	132.74	131.60 ± 5.91
dV/dt_max_ (mV/ms)	114.1	114.80 ± 24.77
APD_80_ (ms)	58.0	58.54 ± 6.16
APD_50_ (ms)	27.0	24.98 ± 3.08

**Fig 10:**
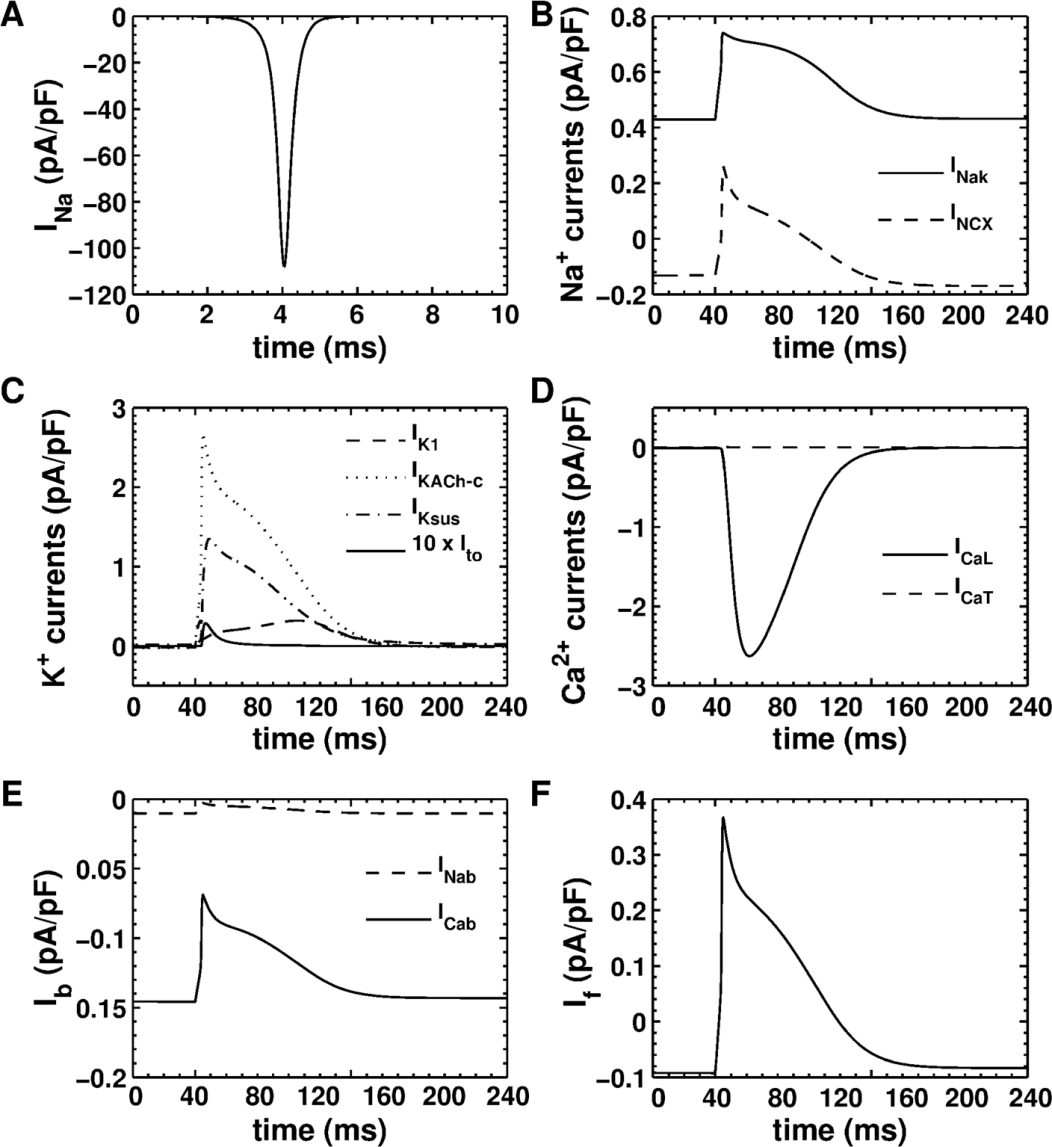
Major ionic currents produced by our model under 1-Hz pacing. **A,** fast Na^+^ current (*I*_*Na*_). **B,** Na^+^/K^+^ pump current (*I*_*NaK*_; solid line) and Na^+^/Ca^2+^ exchanger (*I*_*NCX*_; dashed line). **C,** superposition of all K^+^ currents. **D,** *L-type* (*I*_*CaL*_*)* and *T-type* (*I*_*CaT*_) Ca^2+^ currents. **E,** background Na^+^, and Ca^2+^ currents, (*I*_*Nab*_; dashed line) and (*I*_*Cab*_; solid line), respectively. **F,** hyperpolarization-activated funny current (*I*_*f*_).

Fig 10A–10F show the major ionic currents underlying the AP generated using our single-cell model. The fast Na^+^ current (Fig 10A) generated by our model shows good agreement with the records of Ramos-Mondragόn et al., [35] (*cf*. Fig 4A of Ramos-Mondragόn et al., [35]) Unlike the ventricular model [41], our NRAM model shows reduced *I*_*CaL*_, but comparable *I*_*CaT*_. (Fig 10D) The calculated *I*_*to*_ (Fig 10C) is also much smaller than in the ventricular model [41]. This could explain the absence of the notch in the morphology of the AP. The impure inward rectifier (${\bar{I}}_{K1}$) current in Fig 9C is a composite of the pure *I*_*K1*_ and a K^+^ leakage current.

Overall our model shows strong correspondence of the dynamical behavior of ionic currents with the available direct experimental measurements, and reproduces good integral cell properties such as action potential shape. We provide the numerical code for the single cell model in S1 Code. The next step is to verify wave propagation in two-dimensional (2D) preparations.

### Extension of the single-cell ionic model to higher dimensions (2D monolayers)

#### General formulation

The monodomain formulation was used to extend our single-cell model to higher dimensions. Thus a 2D sheet of cardiac cells, representing a confluent monolayer, was modeled with the following partial differential equation:

$$\frac{\partial V}{\partial t}=\frac{-I_{ion}+I_{stim}}{C_{m}}+\nabla \cdot \left(\bar{D}\nabla V\right),$$ (11)

The second term on the right hand side of equation (Eq 11) takes care of the intercellular coupling. The coupling coefficient $\bar{D}$ is a symmetric, rank-3 tensor, whose elements determine the anisotropy of cardiac tissue. However, ordinary confluent *in vitro* monolayers are isotropic. Therefore, the 2D *in silico* studies presented here, made use of isotropic simulation domains where the diffusion tensor was replaced by a scalar diffusion constant. The temporal part of Eq 11 was integrated using the forward Euler method with time step δt = 0.02 ms, whereas, the spatial part was integrated using a centered finite-differencing scheme with a space step δx = δy = 0.00625 cm and Neumann (zero flux) boundary conditions.

#### Modeling cardiac monolayers

Our *in vitro* monolayers were cultured on circular coverslips in standard 24-well plates which have a basal diameter of 15.6 mm. These cultures showed an average 15–20% randomly-distributed myofibroblasts [11,57–60]. Therefore, to simulate cardiac monolayers that closely resembled our *in vitro* cultures, a circular simulation domain of physical diameter 15.6 mm was used. The myofibroblasts were modeled according to the formulation of MacCannell et al., [61] which treats them as passive circuit elements that are electrically coupled to cardiomyocytes. Simulated cardiomyocytes, together with 15–20% simulated MacCannell-type myofibroblasts [61] were embedded on a rectangular grid, as described previously [22]. Natural intercellular variability was incorporated within the monolayer as described in **Methods**. The cells on the grid were then coupled diffusively (Eq 11). Fig 8A and 8B show a comparison of APs measured at 1Hz electrical pacing using optical mapping, with corresponding voltage traces generated from *in silico* monolayers. The optically mapped APs have similar APDs but aberrant rise and decay times because of filtering associated with the optical mapping technique.

#### Wave propagation in 2D

**Fig 11:**
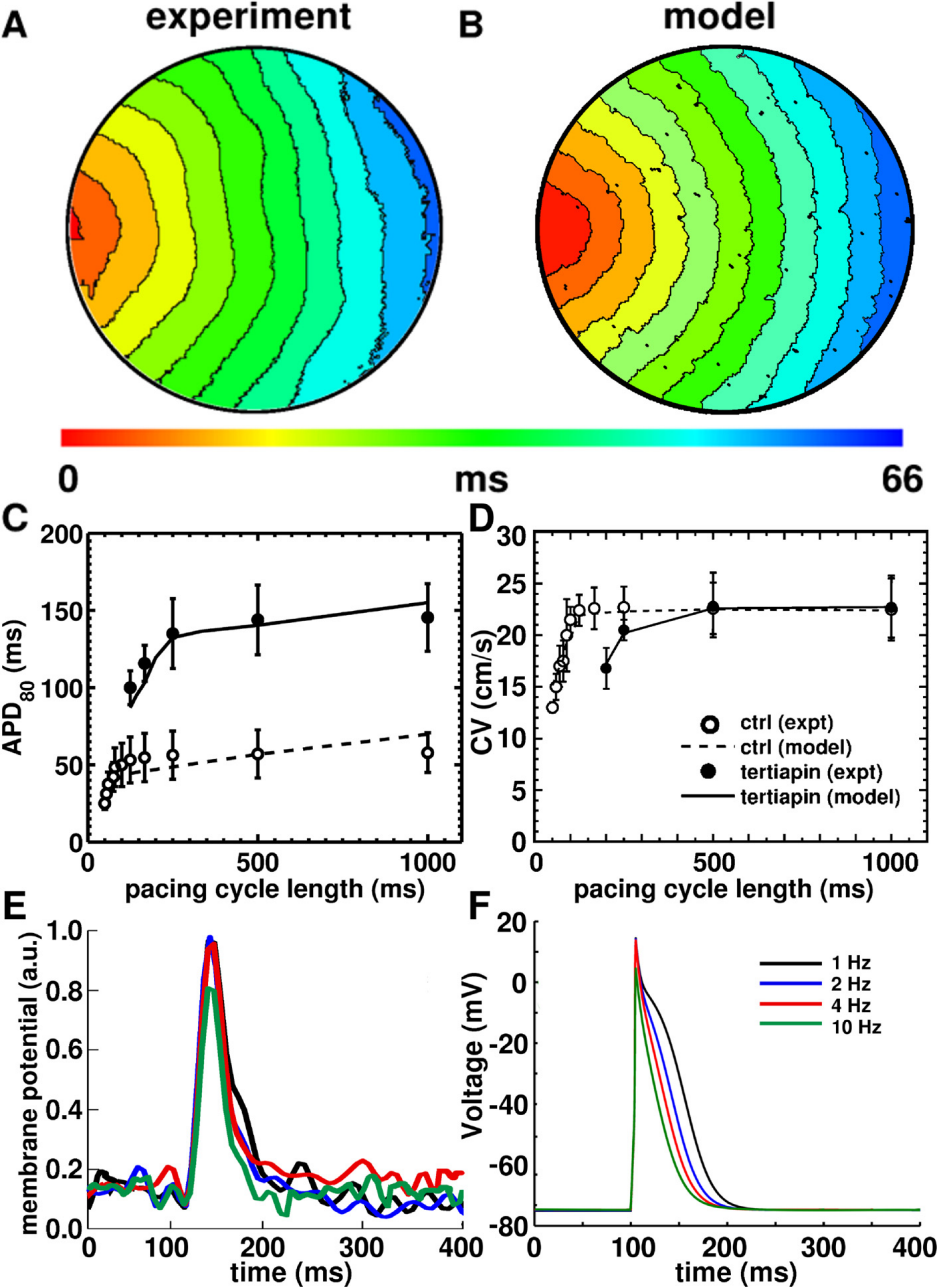
**Activation maps (6 ms isochrone spacing) of NRAM monolayers used for (A) a voltage optical mapping experiment and (B) 2-dimensional (2D) simulations using our baseline monolayer model**. **C,** comparison of conduction velocity restitution (CVR) and (**D**) APD_80_ restitution (APDR) curves generated using our model, with data derived from Bingen et al., [11] for both conditions, in the presence of, and in the absence of the specific Kir3.x-blocker tertiapin-Q. **E**, AP morphologies at pacing frequencies 1Hz, 2Hz, 4Hz and 10Hz (data obtained from voltage optical mapping of NRAM monolayers). **F**, AP morphologies at different pacing frequencies, as generated by our monolayer model.

In monolayer control experiments, NRAMs exhibited a planar conduction velocity (CV) of ~20–25 cm/s. We found that, to obtain such velocity, a diffusion coefficient of 0.00012 cm^2^/ms was required, which resulted in a CV of 22.2 cm/s. Optimal time and space steps for our model were δx = 0.00625 cm, δt = 0.02 ms, which resulted in an error of about 4% for the velocity of wave propagation and was well within tolerable limits [21]. Fig 11A shows baseline electrical propagation in an *in vitro* cardiac monolayer at 1Hz electrical pacing. The corresponding *in silico* activation map generated using our monolayer model is shown in Fig 11B. The two figures show excellent correspondence with roughly the same number of activation contours and similar isochronal line spacing.

In order to develop the model for *I*_*KACh-c*_ in 2D, we fitted the APD_80_ restitution (APDR) curves with experimental data from existing literature. In experiments, both APDR and CVR curves were measured via a dynamic protocol, in which APD_80_ and CV were measured as a function of BCL. Fig 11C shows the APDR curve from a simulated monolayer overlaid on data derived from Fig 5 of Bingen et al. [11] Under control conditions the *in silico* APDR curve corresponds within tolerable limits of the measured data. The simulated curve with blocked *I*_*KACh-c*_ almost exactly coincides with the experimental curve for the tertiapin-Q-treated cell culture. The simulated CV restitution curves (Fig 11D) also show good correspondence with the experimentally measured curves derived from Fig 5 of Bingen et al. [11] The changes in AP morphology in response to electrical pacing at gradually decreasing cycle lengths further demonstrate good agreement between experiment (Fig 11E) and model (Fig 11F).

#### Spiral wave dynamics in the model and experiments

**Fig 12:**
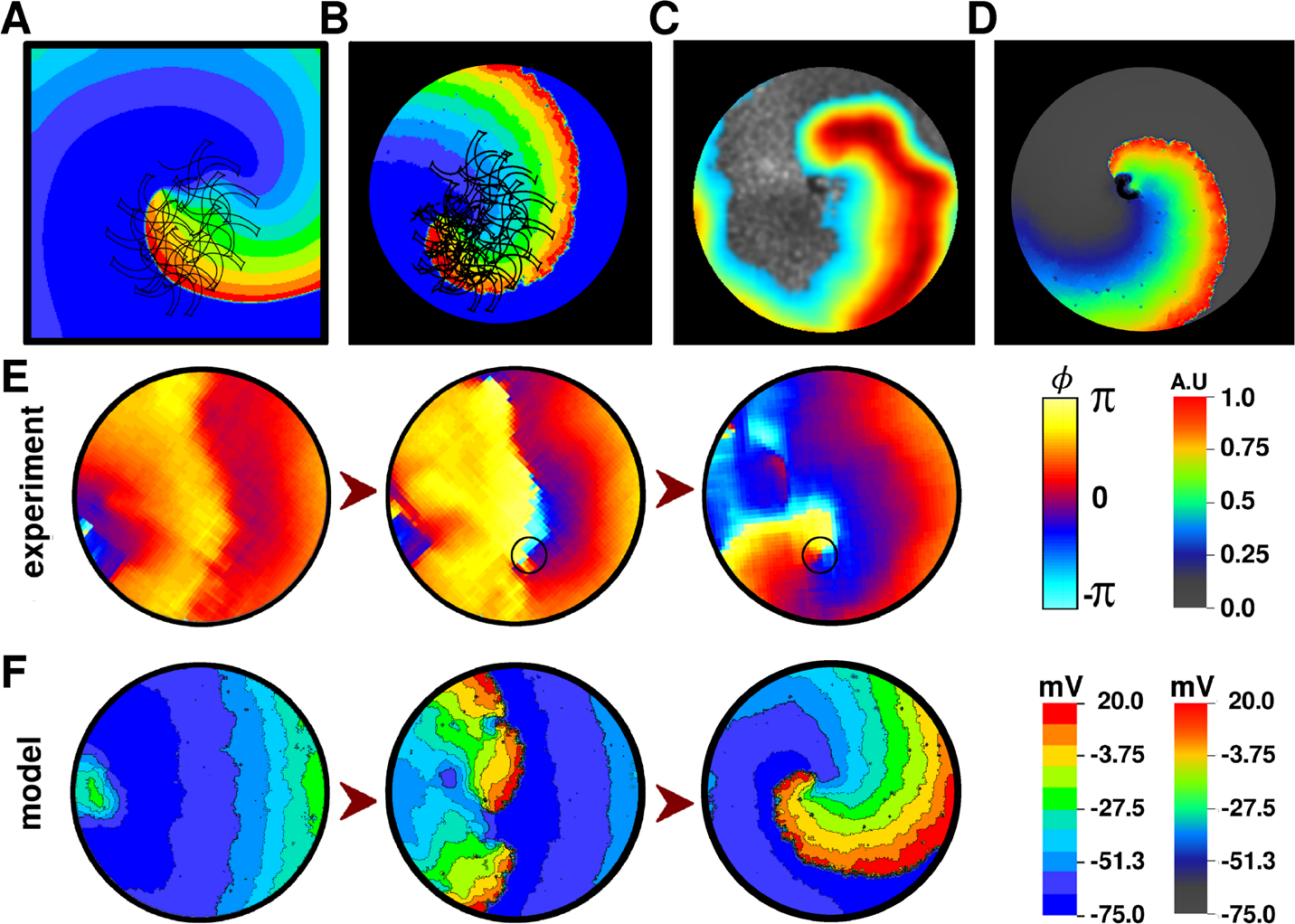
**A, Spiral wave initiated by S1-S2 cross-field protocol in our homogeneous NRAM monolayer model, without myofibroblasts and intercellular variability**. The protocol consisted of applying an S1 stimulus of strength 100 pA and duration 2 ms, along the left border of the simulation domain (x ≤ 5, 0 ≤ y ≤ 256), followed by the application of an S2 stimulus of strength 100 pA and duration 2 ms over the region enclosed by 0 ≤ x ≤ 256 and 162 ≤ y ≤ 256, when the waveback of the first wave crossed half the spatial extent of the simulation domain. Trajectory of the spiral wave is traced in black. **B,** Spiral wave in our heterogeneous monolayer model (containing 17% randomly-distributed myofibroblasts and 50–150% intercellular variability) with tip trajectory (black trace). The protocol for spiral-initiation was same as in **A**. Spiral wave anchored to a clump-type of heterogeneity in the culture dish **C** and in our monolayer model **D,** with geometrically identical myofibroblast heterogeneity. **E,** Phase maps of gradual initiation of reentry upon burst-pacing our *in vitro* monolayer, with open circle to denote the position of the wavebreak. **F,** Voltage maps of burst-pacing induced reentry-initiation in our monolayer model with 17% randomly-distributed myofibroblasts and 50–150% intercellular variability.

Next, we studied spiral wave dynamics in our model. The spiral waves were initiated by two independent protocols: (*i*) the standard S1-S2 cross-field protocol and (*ii*) by burst-pacing. For measurements of the average period of the spiral, the size of the core, and the trajectory traced by the spiral-wave tip, the spiral was generated in a completely homogeneous, square simulation domain, by the S1-S2 cross-field protocol (Fig 12A). The tip of the spiral wave exhibited complex meandering, resulting in a rounded Z-shaped trajectory with a core-size of 5 mm (Fig 12A, black trace). In monolayers with 17% randomly-distributed myofibroblasts and natural cellular heterogeneity, the tip trajectory retained its shape with local irregularities (Fig 12B). The average period of the spiral wave (as estimated from the dominant frequency of the voltage signal) was found to be 80 ± 4.8 ms (model), which is comparable to 81.3±11.3 ms (experiment) [11]. The average APD_80_ during reentry was observed to be 40±4.4 ms (model), which is also comparable to 38.8±7.9 ms (experiment) [11]. Wavelength of the spiral-wave, as calculated from the model (CV * APD_80_ during reentry), was found to be 0.88±0.9 cm, similar to the experimental recording of 0.8±0.2 cm (average value read off Fig 5C of Bingen et al, [11] corresponding to pacing cycle lengths 75–85 ms, which is the range for reentry). In experiments, the tip of the spiral waves were generally non-stationary. They exhibited drift and possibly meander. However, we could not resolve the patterns traced by the tips of the spirals in order to compare with trajectories generated by the model. Therefore, we chose to try and reproduce an experimental situation where the tip of the spiral got anchored to a clump of heterogeneity (Fig 12C). Our monolayer simulations with an identical pattern of myofibroblast heterogeneity also showed anchoring of the spiral wave (Fig 12D). The corresponding wave pattern closely resembled the experimental situation.

To validate the usability of our model in predicting the outcome of *in vitro* experiments, we burst-paced *in silico* monolayers at cycle lengths as low as 80 ms and successfully induced reentry. Fig 12E and 12F show *in vitro* phase maps and representative snapshots of the corresponding *in silico* voltage maps, respectively, of reentry initiation upon burst-pacing.

**Fig 13:**
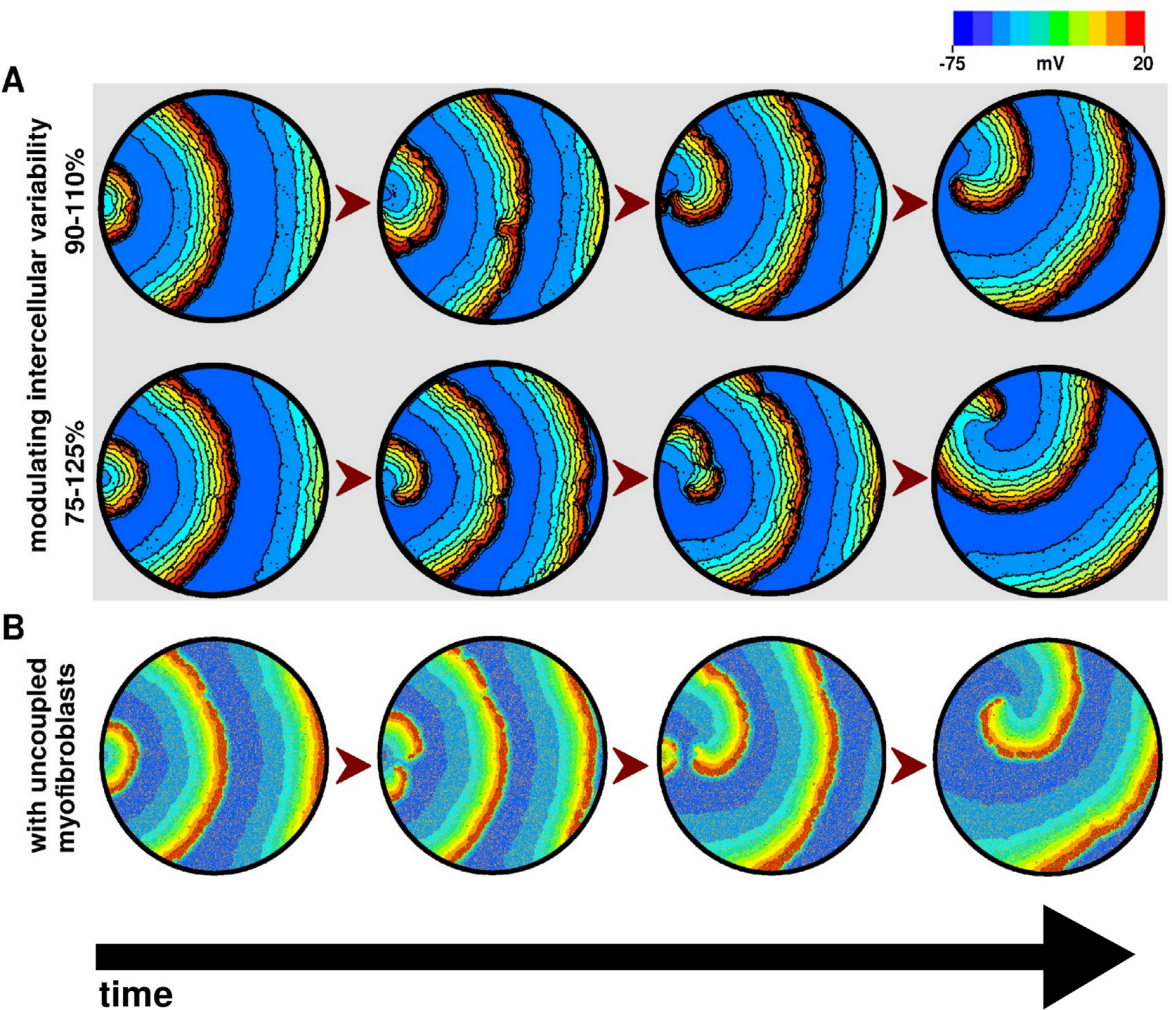
**Gradual initiation of reentry by burst pacing of in silico monolayers containing 17% randomly distributed myofibroblasts and A, 90–110% (upper panel) 75–125% (lower panel) intercellular variability**. **B**, Step-by-step induction of reentry by burst-pacing of a in silico monolayer containing 17% randomly distributed myofibroblasts and 50–150% intercellular variability, but with no coupling between myofibroblasts and cardiomyocytes.

To check whether the specific level of introduced intercellular variability, or the presence of electrotonic coupling with myofibroblasts had any influence on the induction of reentry in our monolayers, we performed 2 separate sets of simulations. In the first set, we used 2 different levels of intercellular variability, namely, 75–125%, and 90–110%, alongside the more commonly used 50–150%. At each variability level, we burst-paced 4 configuration-wise different monolayers and observed 100% stable reentry-induction (Fig 13A). In the second set of experiments, we retained 50–150% intercellular variability but removed the electrotonic coupling between cardiomyocytes and myofibroblasts. This ensured that the myofibroblasts acted like inexcitable obstacles. Burst pacing of these monolayers also led to stable reentry induction in 5 out of 5 trials (Fig 13B).

**Fig 14:**
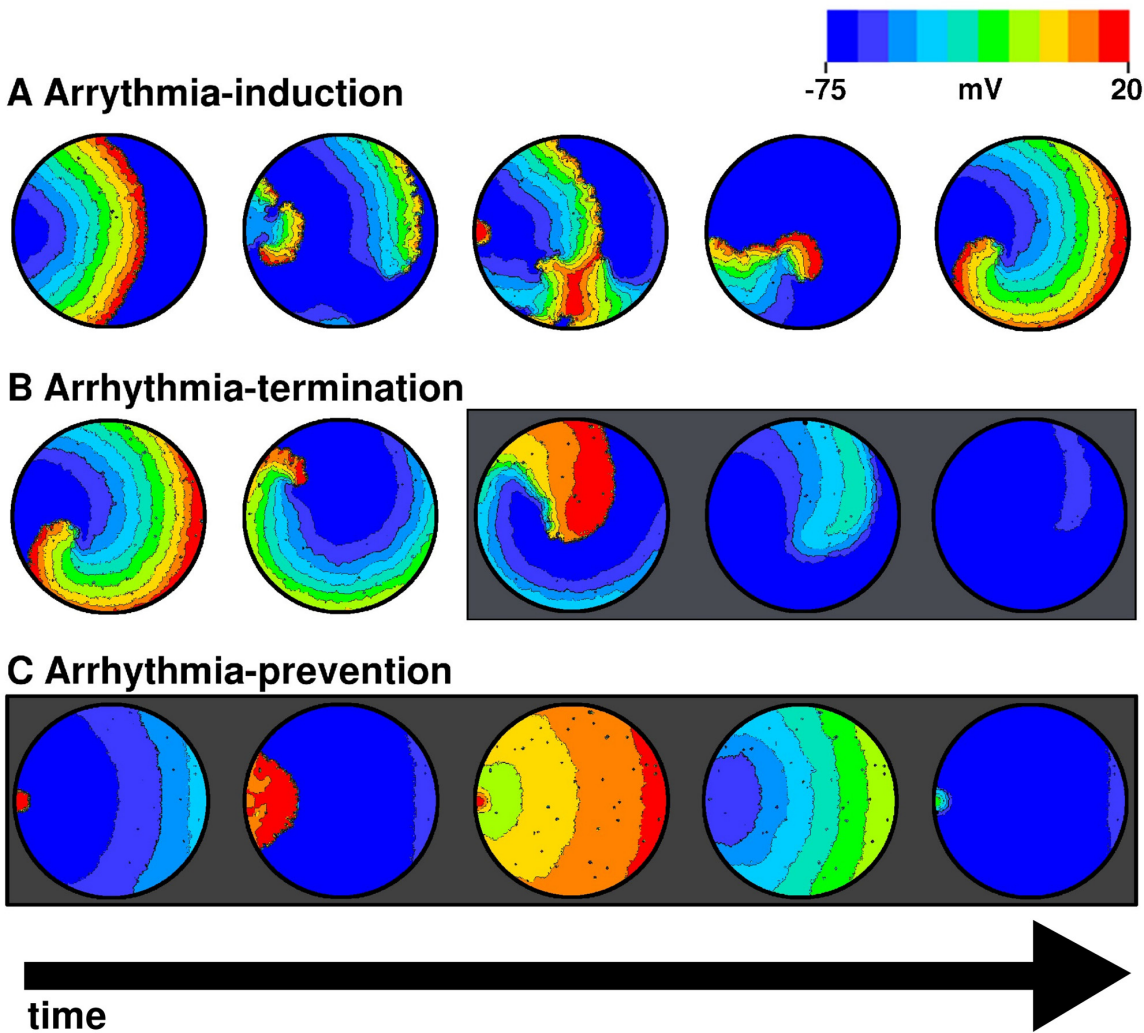
**A, Induction of reentrant activity in a simulated monolayer, by burst pacing**. **B**, termination of spiral waves by complete block of *I*_*KACh-c*_. **C**, prevention of further formation of spirals by burst-pacing, in the absence of *I*_*KACh-c*_. Plots highlighted in gray indicate frames in which *I*_*KACh-c*_ was blocked to mimic the effect of tertiapin-Q.

Finally, to validate the usability of the model in predicting results from in vitro experiments, we used our monolayer model to reproduce our experimental previous results from Bingen et al. [11] Previously, we showed that burst pacing of neonatal rat atrial control monolayers led to robust induction of reentrant arrhythmias. Subsequent block of *I*_*KACh*_ in these monolayers not only led to the removal of existing spirals, but also prevented spiral-formation by burst pacing [11]. Burst-pacing of our monolayer model also showed arrhythmia induction in 10 out of 10 independent cases (Fig 14A). Once an arrhythmia had been induced, we blocked *I*_*KACh*_ completely in our *in silico* monolayers. Complete block of *I*_*KACh*_ resulted in a substantial increase in wavelength through an acute increase in APD. Under the influence of reduced repolarizing currents, the core of the spiral expanded, wavelength increased and the spiral was no longer stable within the confined space of the simulation domain, causing it to disappear (Fig 14B). Finally, burst pacing of the monolayers with completely blocked *I*_*KACh*_ showed that wavebreaks no longer occurred (Fig 14C). The minimum pacing cycle length allowing 1:1 capture of the applied electrical signal shifted to 125 ms, which is in line with the findings of Bingen et al. [11] Therefore, the effect of tertiapin-Q on *I*_*KACh-c*_ could also be reproduced with our monolayer model, at different (75–125% and 90–110%) levels of intercellular variability.

## Discussion

In the present study, we introduced a mathematical model of neonatal rat atrial tissue, based on available experimental electrophysiological data from NRAMs, and our additional patch-clamp data of *I*_*KACh*_ in these cells. If data from NRAMs was unavailable, we used parameters suited to NRVMs. We demonstrated the feasibility of our model to successfully reproduce AP characteristics such as dV/dt_max_, AP amplitude, APD_50_, APD_80_ and RMP.

Using our model, we successfully reproduced the APDR and CVR curves generated using optical mapping experiments on monolayers of neonatal rat atrial cardiomyocytes. These curves are essential determinants of stability of arrhythmias in cardiac tissue.

Furthermore, we demonstrated the feasibility of simulations in spatially extended media with our monolayer model by successfully initiating spiral waves by burst pacing. In agreement with the experiments by Bingen et al., [11] our model demonstrated the possibility of removing reentrant circuits upon inhibition of *I*_*KACh-c*_ by a specific blocker tertiapin-Q. In addition, our model showed a meandering spiral wave with similar average period (80 ± 4.8 ms, versus 81.3 ± 11.3 ms in experiments), and a rounded Z-shaped core of size 5 mm. Using our monolayer model, we also reproduced spiral wave dynamics from our *in vitro* data, in the presence of myofibroblast heterogeneity, thus establishing the robustness of the model, for use alongside experiments.

However, in spite of our best efforts to design a state-of-the-art mathematical model, certain inherent flaws remain. A model is only as good as the data that is used to build it. Since the formulations of most of our currents were derived from patch-clamp data from available literature, we faced the problem of dealing with yet incomplete data extracted using different cell-isolation, cell-culture conditions and the patch-clamp protocols and procedures for obtaining these crucial data. Regardless of the variability, these factors may influence the experimental outcome to a great extent. Therefore, instead of relying on absolute numbers for the maximal currents from the *I-V* curves, we used normalized currents to preserve the trend, while compromising in some cases on the absolute values of the currents produced by the cell.

Discrepancies between results obtained from literature and those derived using our model show up in a few important areas. A major limitation of the model is the lack of evidence on Ca^2+^ dynamics in atrial cardiomyocytes. For simplicity, we adapted the dynamics for intracellular Ca^2+^ from the ventricular model with modifications as described in the S1 Appendix of the supplementary material. However, this lack of key information about Ca^2+^ levels in atrial cardiomyocytes can also perhaps account for the slight alteration in model AP morphology. Such difference may impinge on modeling phenomena where accurate representation of the depolarized state is critical.

Furthermore, our experimental records of *I*_*KACh-c*_-behavior in single cell versus monolayers raises a fundamental question, that of compatibility between single cell and tissue properties. In this respect, it is important to clarify that being 'constitutively active' is not the same as being 'maximally active'. Even a small, but stimulant-independent activity of a current can be regarded as constitutive activity. In our single cell patch-clamp experiments, we observed a high-conductance stimulant-independent current at hyperpolarizing potentials, which gave way to a low-conductance current at physiological depolarized voltages. However, monolayers constructed using the same cells showed a pronounced response to antagonist tertiapin-Q, which translated into 2-fold prolongation of the action potential, indicating the activity of a high-conductance current in monolayers. In 2006, Cha et al, [62] performed dedicated studies to measure *I*_*KACh-c*_ in single and multicellular preparations of canine atrial cardiomyocytes and observed similar anomaly in the behavior of *I*_*KACh-c*_ (see Figs 1C, 1D, 2 and 3 of Cha et al. [62]) Our findings are in line with these observations, i.e., modest versus pronounced effect of tertiapin-Q on APD in single and multicellular level, respectively. One approach to quantify *I*_*KACh-c*_ involves performing patch-clamp studies on neonatal rat atrial cardiomyocytes in the presence of agonist Carbachol. This technique has been used before for that purpose by other researchers such as Dobrev et al. [9] However, addition of Carbachol to observe substantial difference between the IV curves for basal current and *I*_*KACh-c*_ would require (i) an extracellular environment for the cell that contains abnormally high levels of K^+^, resulting in (ii) a condition that will not reflect the actual situation in monolayers, which we aim to model and study *in silico*. To this day, this apparent discrepancy between the behavior of *I*_*KACh-c*_ at single vs multi-cell level remains unresolved. In fact, this matter may not only involve this one particular current, but is likely to concern a more fundamental aspect of single cell vs tissue vs whole organ behavior regarding ion channel dynamics. Therefore, in this manuscript, we employ a reverse-engineering approach in which we use our single cell patch-clamp data for *I*_*KACh-c*_ to build a basal formulation for the *I-V* characteristic curve in our single cell model. When this model is extended to simulate tissue, we combine the concept of Fig 1A from Dobrev et al. [9] about split-*I-V* curves at physiologically relevant depolarized voltages and further adjust this formulation on the basis of control and tertiapin-Q-treated APD restitution curves from monolayer studies, to include the strength of the current at varying pacing cycle lengths. Nevertheless, the translation from a single to multi-cell level in terms of ion channel dynamics certainly warrants further investigation. Until new insights become available, we believe, however, that our study introduces a new and valuable model that is robust enough to facilitate simulation studies into key aspects of atrial fibrillation.

A principal limitation of our model is that an experimentally-validated, detailed Q_10_-compensation was not factored in for usability of this model at all temperatures. Because of the limited availability of experimental data on NRAMs, we used a Q_10_ compensation similar to Hou et al., [51] with certain other adjustments based on the idea that temperature affects channel kinetics of Na^+^, K^+^ and Ca^2+^ currents as well as their maximal conductances, though to a lesser degree. Because no information was available regarding any of the time constants for the gating variables that controlled the different ionic currents, except fast Na^+^ activation, all the time constants were either taken from the neonatal rat ventricular cardiomyocyte model by Korhonen et al., [41] or adapted to suit the AP characteristic and APDR data fromNRAMs.

Inactivation kinetics of the fast Na^+^ channel is an important determinant of cardiac tissue excitability. In our model, we fitted the steady-state inactivation curve with data derived from Voitychuk et al. [36] The V_½_ for this data was ~15 mV more positive than in the steady-state inactivation data of Ramos-Mondragón et al. [35] However, we chose to use the data derived from Voitychuk et al., [36] instead of the data derived from Ramos-Mondragón et al., [35] because for a fixed *dV/dt*_*max*_ the kinetics from Ramos-Mondragón et al, did not allow wave propagation in a monolayer, as opposed to the kinetics from Voitychuk et al. [36]

Taken together, we present in this paper, a quantitatively-robust, computationally-efficient, experimentally-validated mathematical model of the NRAM expressing the *I*_*KACh-c*_. We use this model to build simulation domains that mimic cardiac monolayers *in vitro* and successfully demonstrate the usability of the monolayer model to study initiation, maintenance and termination of spiral wave arrhythmias by reproducing key results from our previous *in vitro* experiments.

## Methods

Animal protocols were reviewed and approved by the Animal Experiments Committee of the Leiden University Medical Center (LUMC) and conformed to the Guide for the Care and Use of Laboratory Animals as stated by the US National Institute of Health.

### Cell isolation and culturing

NRAMs were isolated from hearts of 2-day-old Wistar rat pups as previously described [11,63]. Isolated cells were plated on round glass coverslips (15-mm diameter) coated with fibronectin (Sigma-Aldrich, St. Louis, MO, USA) in 24-well plates (Corning Life Sciences, Amsterdam, the Netherlands). Depending on the assay, cell densities of 0.1–8 x 10^5^ cells/well were used and culture as described in our commonly used neonatal rat cardiac myocyte preparation [57,58].

### Patch-clamp electrophysiology

NRAMs were patch-clamped using the whole-cell technique at 20–23°C on day 2–4 of culture. The patch pipettes were pulled from borosilicate glass capillaries (1.5 mm outer diameter and 1.17 mm inner diameter; Harvard Apparatus, Kent, UK) with a vertical puller (P-30; Sutter Instrument Company, Novato, CA, USA). Pipettes had typical electrical resistances of 2–3 MΩ in the extracellular solution when filled with the internal solution (for composition, see later). Solitary beating cells were selected under an inverted microscope Zeiss Axiovert 35 (Carl Zeiss AG, Oberkochen, Germany) for the measurements by a MultiClamp 700B amplifier connected to a Digidata 1440A A/D converter (Axon CNS, Molecular Devices, Sunnyvale, CA, USA). After reaching the giga-ohm seal, the holding potential was set to -50 mV, and the patch membrane was ruptured by gentle suction through the pipette. Whole-cell capacitance (C_m_) was calculated from capacitive transient currents evoked during 5 mV steps from a holding potential of -50 mV. The capacitance transients were cancelled with the amplifier. To minimize voltage error and improve the adequacy of the voltage-clamp, pipette series resistance was routinely electrically compensated by >75%. The voltage and current command pulses were applied to the amplifier through its external command input from the A/D converter connected to a personal computer. The instruments were controlled and driven by commercially available MultiClamp 700B Commander and Clampex V10.3 softwares (Molecular Devices) for Windows. Throughout experiments, the current and voltage outputs of amplifier were continuously sampled at intervals of 100 μs and recorded onto the personal computer after low-pass filtering at 2–4 kHz with a four-pole Bessel filter.

### Solutions

Action potentials and total membrane currents were recorded in the standard extracellular solution containing (in mM): 126 NaCl, 11 glucose, 10 HEPES, 5.4 KCl, 1 MgCl_2_, and 1.8 CaCl_2_ (adjusted to pH 7.40 with NaOH). The standard internal pipette solution used for recording of action potentials and total membrane currents contained (in mM): 80 potassium DL-aspartate, 40 KCl, 8 NaCl, 5.5 glucose, 5 HEPES, 5 EGTA, 1 MgCl 2, 4 Mg-ATP, and 0.1 Na 3 -GTP (adjusted to pH 7.20 with KOH). To study the effect of *I*_*KACh*_ on AP, 2 μM carbachol (Sigma-Aldrich, St Louis, MO, USA) and 100 nM tertiapin-Q (Alomone Labs, Jerusalem, Israel) were added to standard extracellular solution to activate and block this current in NRAMs, respectively A correction of approximately 11 mV, because of liquid junction potential was applied in the analysis of action potential recordings.

Time-dependent hyperpolarization-activated currents (*I*_*KH*_) were recorded in a modified extracellular solution. In addition to the standard extracellular solution, tetrodotoxin (30 μM) (Alomonne Labs), nitrendipine (10 μM), 4-Aminopyridine (2mM) and atropine (100 nM) all from Sigma-Aldrich were added in this solution to suppress, *I*_*Na*_, *I*_*CaL*_, *I*_*to*_ and muscarinic receptor-activated currents, respectively. The highly selective Kir3.X channel blocker tertiapin-Q (100 nM) was used to isolate *I*_*K1*_ from the total *I*_*KH*_. The standard internal pipette solution used for the experiments contained (in mM): 80 potassium DL-aspartate, 40 KCl, 8 NaCl, 5.5 glucose, 5 HEPES, 5 EGTA, 1 MgCl 2, 4 Mg-ATP, and 0.1 Na 3 -GTP (adjusted to pH 7.20 with KOH)was used to fill the pipettes.

### Data analysis and statistics

For off-line analysis, the data stored on the personal computer were analyzed by pClamp V10.3 (Molecular Devices) and GraphPad Prism software version 6 (GraphPad Software, Inc., La Jolla, CA, USA). All data are expressed as means ± standard error of mean (SEM) for a specified number (n) of observations. Significant differences were determined at the P <0.05 level, unless specified. Unpaired Student’s t test or the one-way ANOVA test followed by Tukey’s test were used for comparing different experimental groups. Statistical significance was expressed as follows: *: P<0.05, **: P<0.01, ***: P<0.001 NS: not significant.

### Construction of virtual monolayers

Virtual monolayers were constructed by allocating cells on a circular selection in a simulation domain containing 256 x 256 grid points. In order to achieve a random distribution of myofibroblasts within the monolayers, N specific sites were selected from within the array, by a random-number generator. For consistency with experiments, N was chosen such that the myofibroblasts constituted 15–20% of the total number of cells comprising the monolayers. The myofibroblasts were coupled to the cardiomyocytes via a gap junctional conductance G_gap_ = 0.5 nS/pF [59]. This coupling is ~6 times smaller than myocyte-myocyte coupling. Whole-cell patch-clamp measurements of APs from different samples of NRAMs showed a slight variation in AP characteristics (S1 Fig). When cardiomyocytes as heterogeneous as these are coupled together to form a monolayer, it would therefore be reasonable to expect a certain degree of natural intercellular variability within the monolayer, although most of the heterogeneity in electrophysiological properties would be compensated by electrotonic coupling. Please refer to Fig 7D for proof of variability in the conductance values of currents *I*_*K1*_ and *I*_*KACh*_. It is possible to adopt a very basic approach to accomplish the same in computer models. To incorporate X_1_-X_2_% intercellular variability within the monolayers the following protocol was used: (*i*) A random-number generator was used to generate 19 numbers from within the range 0.01 x (X_1_-X_2_), for each cardiomyocyte occupying a site on the cellular grid. (*ii*) At each cell site, the maximal conductance, associated with an ionic current/flux, was multiplied by one of these 19 random numbers (one random number for each individual ionic current/flux). This ensured a spatially-random distribution between the electrophysiological properties of the individual uncoupled cells. Next, these heterogeneous cells were coupled electrotonically. Electrotonic coupling averages out the electrophysiological properties so that the culture exhibits uniform APD distributions. Simulated monolayers were discarded if the APD_80_ maps showed non-uniform distribution of APD or APD dispersion larger than 20 ms. Finally, APD_80_ maps from our *in vitro* cultures were compared with those from the simulated monolayers with varying levels of intercellular heterogeneity. Because the APD_80_ maps at 50–150% intercellular variability showed closest resemblance with *in vitro* data in terms of mean value and spatial distribution, we chose to use this range for our numerical experiments.

## Supporting Information

S1 Appendix (DOCX)

S1 Fig **A,** Intercellular variability in the patch clamp recordings of 4 APs recorded from 4 different neonatal rat atrial cardiomyocytes, cultured under the same normal growth conditions, in the control environment. **B,** RMP maps obtained from simulated monolayers with varying levels of intercellular variability. Mean RMP is ~-73 mV in each case with progressively increased dispersion, as range of variability becomes more widespread (left to right). **C,** A representative APD_80_ map from our *in vitro* control monolayers. D, APD_80_ maps measured from *in silico* monolayers, corresponding to the RMP maps presented in the upper panel.
(TIFF)

S1 Code Code_for_Atrial_model.tar.gz. (GZ)
