# Supplementary material for: A Mathematical Model of Neonatal Rat Atrial Monolayers with Constitutively Active Acetylcholine-Mediated K+ Current
Source: PLoS Comput Biol. 2016 Jun 22;12(6):e1004946. doi: 10.1371/journal.pcbi.1004946 (PMC4917258; doi:10.1371/journal.pcbi.1004946)
Supplement: S1 Appendix — (DOCX) [file pcbi.1004946.s001.docx]

**S1 Appendix**

Membrane potential

$\frac{\partial V}{\partial t}=\nabla\cdot\left( \tilde{D}\nabla V \right)-\frac{I_{ion}+I_{stim}}{C_{m}},$ (1)

 $I_{ion}=I_{CaL}+I_{CaT}+I_{NCX}+I_{Cab}+I_{Nab}+I_{NaK}+I_{Na}+I_{f}+I_{Ksus}+I_{K1}+I_{to}+I_{K,ACh-c},$ (2)

The temporal part of Eq.1 was integrated using the forward Euler method with time step δt=0.02 ms. In the formulations for the time-dependent ionic currents, the gating variables obeyed Hodgkin-Huxley-type equations. These were integrated using the Rush and Larsen scheme [1].

Fast Na^+^ current

$E_{Na}=\frac{RT}{F}ln\left( \frac{\left[ Na \right]_{o}}{\left[ Na \right]_{i}} \right),$ (3)

$I_{Na}=0.9G_{Na}m^{3}hj\left( V-E_{Na} \right),$ (4)

$\alpha_{m}=0.32\frac{\left( V+47.13 \right)}{\left( 1-e^{-0.1\left( V+47.13 \right)} \right)},$ (5)

$\beta_{m}=0.056e^{\left( \frac{-V}{11} \right)},$ (6)


If $V<-40mV$

$\alpha_{h}=0.135e^{\frac{-\left( V+70.0 \right)}{6.8}},$ (7)

$\beta_{h}=3.56e^{0.079V}+3.1\times{10}^{5}e^{0.35V},$ (8)

$\alpha_{j}=2.0\left( -1.2714\times{10}^{5}e^{0.2444V}-3.474\times{10}^{-5}e^{-0.04391V} \right)\left( \frac{V+37.78}{1+e^{0.311\left( V+79.23 \right)}} \right),$ (9)

$\beta_{j}=0.1212\frac{e^{-0.01052V}}{1+e^{-0.1378\left( V+40.14 \right)}},$ (10)

otherwise $\alpha_{h}=0.0,$ (11)

$\beta_{h}=\frac{1.0}{0.13\left( 1+e^{\left( -\frac{V+10.66}{11.1} \right)} \right)},$ (12)

$\alpha_{j}=0.0,$ (13)

$\beta_{j}=\frac{0.3e^{\left( -2.535\times{10}^{-7}V \right)}}{1.0+e^{-0.1\left( V+32.0 \right)}},$ (14)

$m_{\infty}=\frac{1.0}{1.0+e^{\left( \frac{37.0+V}{-6.8} \right)}},$ (15)

$\tau_{m}=\frac{1.0}{\alpha_{m}+\beta_{m}}$ (16)

$h_{\infty}=\frac{1.0}{1.0+e^{\left( \frac{80.0+V}{9.2} \right)}},$ (17)

If $V<-40mV$

$\tau_{h}=\frac{0.06}{\alpha_{h}+\beta_{h}}+1.5,$ (18)

otherwise

$\tau_{h}=\frac{0.75}{\alpha_{h}+\beta_{h}}+0.15,$ (19)

$\tau_{j}=\frac{1.0}{\alpha_{j}+\beta_{j}},$ (20)

$j_{\infty}=h_{\infty},$ (21)

$\frac{dm}{dt}=\frac{m_{\infty}-m}{\tau_{m}},$ (22)

$\frac{dh}{dt}=\frac{h_{\infty}-h}{\tau_{h}},$ (23)

$\frac{dj}{dt}=\frac{j_{\infty}-j}{\tau_{j}},$ (24)

Hyperpolarization activated current

$I_{fNa}=G_{f}y\left( 0.2\left( V-E_{Na} \right) \right),$ (25)

$I_{fK}=G_{f}y\left( 0.8\left( V-E_{K} \right) \right),$ (26)

$I_{f}=I_{fNa}+I_{fK},$ (27)

$y_{\infty}=\frac{1.0}{1+e^{\left( \frac{V+78.65}{6.33} \right)}},$ (28)

$\tau_{y}=\frac{1000.0}{0.11885e^{\left( \frac{V+75}{28.37} \right)}+0.56236e^{\left( \frac{V+75}{-14.19} \right)}},$ (29)

$\frac{dy}{dt}=\frac{y_{\infty}-y}{\tau_{y}},$ (30)

Sustained outward K^+^ current

$I_{Ksus}=0.001\left( I_{Kur}+I_{Ks}+I_{Kr} \right),$ (31)

$E_{K}=\frac{RT}{F}ln\left( \frac{\left[ K \right]_{o}}{\left[ K \right]_{i}} \right),$ (32)

$E_{Ks}=\frac{RT}{F}ln\left( \frac{\left[ K \right]_{o}+P_{NaK}\left[ Na \right]_{o}}{\left[ K \right]_{i}+P_{NaK}\left[ Na \right]_{i}} \right),$ (33)

 Ultra-rapid outward K^+^ current

$I_{Kur}=G_{Kur}u_{a}u_{i}\left( V-E_{K} \right),$ (34)

$u_{a\infty}=\frac{1.0}{1+e^{\left( -\frac{V+12.5}{25} \right)}},$ (35)

$\tau_{ua}=0.493e^{-0.0629V}+2.058,$ (36)

$u_{i\infty}=\frac{1.0}{1+e^{\left( \frac{V-100}{5.7} \right)}},$ (37)

$\tau_{ui}=1200-\frac{170.0}{1+e^{\left( \frac{V+45.2}{5.7} \right)}},$ (38)

$\frac{{du}_{a}}{dt}=\frac{u_{a\infty}-u_{a}}{\tau_{ua}},$ (39)

$\frac{{du}_{i}}{dt}=\frac{u_{i\infty}-u_{i}}{\tau_{ua}},$ (40)

Slow delayed rectifier K^+^ current

$G_{Ks}=0.0866\left( 1+\frac{0.6}{1+\left( \frac{0.000038}{\left[ Ca \right]_{i}} \right)^{1.4}} \right),$ (41)

$I_{Ks}=G_{Ks}{Xs}_{1}{Xs}_{2}\left( V-E_{Ks} \right),$ (42)

${Xs}_{1\infty}=\frac{1.0}{1+e^{\left( -\frac{V-10}{20} \right)}},$ (43)

${Xs}_{2\infty}={Xs}_{1\infty},$ (44)

$\tau_{Xs1}=\frac{1.0}{7.19\times{10}^{-5}\frac{\left( V+30 \right)}{1-e^{-0.148\left( V+30 \right)}}+1.31\times{10}^{-4}\frac{\left( V+30 \right)}{e^{0.0687\left( V+30 \right)}-1}},$ (45)

$\tau_{Xs2}=4\tau_{Xs1,}$ (46)

$\frac{{dXs}_{1}}{dt}=\frac{{Xs}_{1\infty}-{Xs}_{1}}{\tau_{Xs1}},$ (47)

$\frac{{dXs}_{2}}{dt}=\frac{{Xs}_{2\infty}-{Xs}_{2}}{\tau_{Xs2}},$ (48)


Rapid delayed rectifier K^+^ current

$G_{Kr}=0.0005228\sqrt{\left( \frac{\left[ K \right]_{o}}{5.4} \right)},$ (49)

$I_{Kr}=G_{Kr}X_{r}R_{r}\left( V-E_{K} \right),$ (50)

${Xr}_{\infty}=\frac{1.0}{1+e^{\left( -\frac{V+12.5}{10} \right)}},$ (51)

$\tau_{Xr}=\frac{1.0}{\frac{0.00138\left( V+14.2 \right)}{1-e^{-0.123\left( V+14.2 \right)}}+\frac{0.00061\left( V+38.9 \right)}{e^{0.145\left( V+38.9 \right)}-1}},$ (52)

${Rr}_{\infty}=\frac{1.0}{1+e^{\left( \frac{V+9.0}{22.4} \right)}},$ (53)

$\frac{dXr}{dt}=\frac{{Xr}_{\infty}-Xr}{\tau_{Xr}},$ (54)

Time-independent K^+^ current

$\bar{I}_{K1}=0.0489\left( \frac{K_{o}}{K_{o}+210} \right)\left( \frac{V-E_{K}-10}{1.0+e^{0.041\left( V-E_{K}-10 \right)}} \right)+0.01\left( V-E_{K} \right),$ (55)


Transient outward K^+^ current

$I_{to}=G_{t}r\left( 0.706s+0.294s_{slow} \right)\left( V-E_{K} \right),$ (56)

$r_{\infty}=\frac{1.0}{1+e^{\left( -\frac{V+3}{12} \right)}},$ (57)

$s_{\infty}=\frac{1.0}{1+e^{\left( \frac{V+31.97156}{4.64291} \right)}},$ (58)

$s_{slow\infty}=s_{\infty},$ (59)

$\tau_{r}=\frac{1000.0}{45.16e^{0.03577\left( V+50 \right)}+98.9e^{-0.1\left( V+38.0 \right)}},$ (60)

$\tau_{s}=1000\left( 0.35e^{-\left( \frac{V+70}{15} \right)^{2}}+0.035 \right)-26.9,$ (61)

$\tau_{sslow}=1000\left( 3.7e^{-\left( \frac{V+70}{30} \right)^{2}}+0.035 \right)+37.4,$ (62)

$\frac{dr}{dt}=\frac{r_{\infty}-r}{\tau_{r}},$ (63)

$\frac{ds}{dt}=\frac{s_{\infty}-s}{\tau_{s}},$ (64)

$\frac{{ds}_{slow}}{dt}=\frac{s_{slow\infty}-s_{slow}}{\tau_{sslow}},$ (65)

Na^+^/K^+^ ATPase

$\sigma=\frac{\left( e^{\left( \frac{\left[ Na \right]_{o}}{67300} \right)}-1 \right)}{7,}$ (66)

$f_{NaK}=\frac{1.0}{1+0.1245e^{\left( -\frac{0.1VF}{RT} \right)}+0.0365\sigma e^{\left( -\frac{VF}{RT} \right)}},$ (67)

$I_{NaK}=\bar{I}_{NaK}\frac{f_{NaK}}{\left( 1+\left( \frac{K_{mNai}}{\left[ Na \right]_{i}} \right)^{n_{NaK}} \right)\left( 1+\frac{K_{mKo}}{\left[ K \right]_{o}} \right)},$ (68)


Constitutively active K,ACh current

$I_{KACh}=\left( 0.296 \right)\left( 0.1+\frac{0.18}{1.0+e^{\left( \frac{V+102.0}{10.0} \right)}} \right)\left( V-E_{K}-10 \right),$ (69)

For SL membrane currents

L-type Ca^2+^ current

$E_{Ca}=\frac{RT}{2F}ln\left( \frac{\left[ Ca \right]_{o}}{\left[ Ca \right]_{i}} \right),$ (70)

$I_{CaL}=4G_{CaL}dff_{Ca}\frac{{VF}^{2}}{RT}\left( \frac{\left[ Ca \right]_{i}e^{\left( \frac{2VF}{RT} \right)}-0.341\left[ Ca \right]_{o}}{e^{\left( \frac{2VF}{RT} \right)}-1} \right),$ (71)

$d_{\infty}=\frac{1.0}{1+e^{\left( \frac{-1.8-V}{8.6} \right)}},$ (72)

$a_{d}=0.25+\frac{1.4}{1.0+e^{\left( \frac{-35-V}{13} \right)}},$ (73)

$b_{d}=\frac{1.4}{1+e^{\left( \frac{V+5}{5} \right)}},$ (74)

$c_{d}=\frac{1.0}{1+e^{\left( \frac{50-V}{20} \right)}},$ (75)

$\tau_{d}=a_{d}b_{d}+c_{d}+10.0,$ (76)

$f_{\infty}=\frac{1.0}{1+e^{\left( \frac{22+V}{6.1} \right)}},$ (77)

$\tau_{f}=562.5e^{-\frac{\left( V+27.0 \right)^{2}}{1000}}+\frac{10.0}{1+e^{\left( 25-V \right)}}+10.0,$ (78)

$a_{fCa}=\frac{1.0}{1+\left( \frac{\left[ Ca \right]_{i}}{0.325} \right)^{8}},$ (79)

$b_{fCa}=\frac{0.1}{1+e^{\left( \frac{\left[ Ca \right]_{i}-0.5}{0.1} \right)}},$ (80)

$c_{fCa}=\frac{0.2}{1+e^{\left( \frac{\left[ Ca \right]_{i}-0.75}{0.8} \right)}},$ (81)

${fCa}_{\infty}=\frac{a_{fCa}+b_{fCa}+c_{fCa}+0.23}{1.46},$ (82)

$k=\left( \begin{aligned} 0, \\ 1, \end{aligned}\begin{aligned} {fCa}_{\infty}>f_{Ca},V>-60mV \\ otherwise \end{aligned} \right),$ (83)

$\frac{dd}{dt}=\frac{d_{\infty}-d}{\tau_{d}},$ (84)

$\frac{df}{dt}=\frac{f_{\infty}-f}{\tau_{f}},$ (85)

$\frac{{df}_{Ca}}{dt}=k\frac{f_{Ca\infty}-f_{Ca}}{\tau_{fCa}},$ (86)

T-type Ca^2+^ current

$I_{CaT}=G_{CaT}bg\left( V-E_{Ca}+106.5 \right),$ (87)

$b_{\infty}=\frac{1.0}{1+e^{\left( -\frac{V+36}{6.1} \right)}},$ (88)

$\tau_{b}=0.6+\frac{5.4}{1+e^{0.03\left( V+100 \right)}},$ (89)

$g_{\infty}=\frac{1.0}{1+e^{\left( \frac{V+66}{6} \right)}},$ (90)

$\tau_{g}=1+\frac{40.0}{1+e^{0.08\left( V+65 \right)}},$ (91)

$\frac{db}{dt}=\frac{b_{\infty}-b}{\tau_{b}},$ (92)

$\frac{dg}{dt}=\frac{g_{\infty}-g}{\tau_{g}},$ (93)

Na^+^/Ca^2+^ exchanger current

$I_{NCX}=k_{NCX}\frac{{\left[ Na \right]_{i}}^{3}\left[ Ca \right]_{o}e^{0.03743\gamma V}-{\left[ Na \right]_{o}}^{3}\left[ Ca \right]_{i}e^{0.03743\left( \gamma-1 \right)V}}{1+d_{NCX}\left( {\left[ Na \right]_{o}}^{3}\left[ Ca \right]_{i}+{\left[ Na \right]_{i}}^{3}\left[ Ca \right]_{o} \right)},$ (94)

Background Ca^2+^ and Na^+^ currents

$I_{Cab}=G_{Cab}\left( V-E_{Ca} \right),$ (95)

$I_{Nab}=G_{Nab}\left( V-E_{Na} \right),$ (96)


Calcium fluxes

$J_{rel}=\nu_{1}P_{o1}\left( \left[ Ca \right]_{JSR}-\left[ Ca \right]_{i} \right),$ (97)

$J_{leak}=k_{leak}\left( \left[ Ca \right]_{NSR}-\left[ Ca \right]_{i} \right),$ (98)

$J_{tr}=\left( \frac{\left[ Ca \right]_{NSR}-\left[ Ca \right]_{JSR}}{\tau_{tr}} \right),$ (99)

$\frac{d{Ca}_{NSR}}{dt}=\frac{J_{up}-J_{leak}-J_{tr}}{V_{NSR}}$ (100)

$\frac{d{Ca}_{JSR}}{dt}=\beta_{SR}\frac{-J_{rel}+J_{tr}}{V_{JSR}},$ (101)

Free intracellular Ca^2+^

$\beta_{Cai}=\frac{1.0}{\left( 1+\frac{\overline{TRPN}K_{mTRPN}}{\left( \left[ Ca \right]_{i}+K_{mTRPN} \right)^{2}}+\frac{\overline{CMDN}K_{mCMDN}}{\left( \left[ Ca \right]_{i}+K_{mCMDN} \right)^{2}} \right)},$ (102)

$\frac{d\left[ Ca \right]_{i}}{dt}=\beta_{Cai}\left( \frac{J_{CaSR}+J_{CaSL}}{V_{myo}} \right),$ (103)

Ca^2+^ buffering

$\beta_{SR}=\frac{1.0}{\left( 1+\frac{\overline{CSQN}K_{mCSQN}}{\left( \left[ Ca \right]_{JSR}+K_{mCSQN} \right)^{2}} \right)},$ (104)

Ryanodine receptor gating

$K_{mRyR}=\frac{3.51}{1.0+e^{\left( \frac{\left[ Ca \right]_{JSR}-530}{200} \right)}}+0.25,$ (105)

$P_{C1}=1-P_{o1,}$ (106)

$\frac{{dP}_{o1}}{dt}=k_{a}\left( \frac{{\left[ Ca \right]_{i}}^{N}}{{\left[ Ca \right]_{i}}^{N}+{K_{mRyR}}^{N}} \right)P_{C1}-k_{b}P_{o1},$ (107)

SERCA

$J_{up}=\frac{V_{max}\left( \frac{\left[ Ca \right]_{i}}{K_{mf}} \right)^{H_{f}}-V_{max}\left( \frac{\left[ Ca \right]_{NSR}}{K_{mr}} \right)^{H_{r}}}{1+\left( \frac{\left[ Ca \right]_{i}}{K_{mf}} \right)^{H_{f}}+\left( \frac{\left[ Ca \right]_{NSR}}{K_{mr}} \right)^{H_{r}}},$ (108)

$J_{CaSR}=J_{rel}-J_{up}+J_{leak},$ (109)

$J_{CaSL}=\left( 2I_{NCX}-I_{CaL}-I_{CaT}-I_{Cab} \right)\frac{A_{cap}C_{m}}{2F\times{10}^{-6}},$ (110)

Temperature scaling in monolayer model:

$h_{\infty,310K}=\frac{1.0}{1.0+e^{\left( \frac{78.0+V}{7.8} \right)}},$ (111)

Furthermore, $\tau_{X,310K}=\frac{\tau_{X,296K}}{Q_{10}}$ for $X\in\left( m,h,j,d,f,fCa,ua,ui,Xs1,Xs2,Xr \right)$, and $G_{Na,310K}=1.07G_{Na,296K}$.

**Model constants:**

| Parameter | Description | Value |
| --- | --- | --- |
| *radius*  *F*  *R*  *T*  *C_m_*  *Q_10_*  *r_nucleus_*  *r_SR_*  *r_SL_*  *A_cap_*  *V_SR_*  *V_NSR_*  *V_JSR_*  *V_myo_*  [Ca]_o_  [Na]_o_  [K]_o_  [Na]_i_  [K]_i_  *G_CaL_*  *G_CaT_*  *G_Cab_*  *G_Nab_*  *G_Kb_*  *G_Na,_*_310K_  *G_Kur_*  *G_f_*  *G_to_*  *k_NCX_*  *d_NCX_*  *γ*  *I_NaK_*  *K_mNai_*  *n_NaK_*  *K_mKo_*  *τ_fCa_*  *P_NaK_*  *ν_1_*  *V_max_*  *K_mf_*  *K_mr_*  *H_f_*  *H_r_*  *k_leak_*  *τ_tr_*    *TRPN*  *K_mTRPN_*  *CMDN*  *K_mCMDN_*  *CSQN*  *K_mCSQN_*    k_a_  k_b_  *N* | Radius of petridish  Faraday's constant  Ideal gas constants  Temperature  Specific membrane capacitance  Temperature scaling coefficient  Radius to the surface of nucleus  Radius to the surface of SR  Radius of the cell  Capacitive membrane area  Volume of sarcoplasmic reticulum  Volume of junctional sarcoplasmic reticulum  Volume of network sarcoplasmic reticulum  Volume of myoplasm  Extracellular Ca^2+^ concentration  Extracellular Na^+^ concentration  Extracellular K^+^ concentration  Intracellular Na^+^ concentration  Intracellular K^+^ concentration  Maximum *I_CaL_* conductance  Maximum *I_CaT_* conductance  *I_Cab_* conductance  *I_Nab_* conductance  Maximum *I_Kb_* conductance  Maximum *I_Na_* conductance at 310K  Maximum *I_Kur_* conductance  Maximum *I_f_* conductance  Maximum *I_t_* conductance  Scaling factor *I_NCX_*  Denominator constant for *I_NCX_*  Energy barrier parameter for *I_NCX_*  Maximum NaK-ATPase current  Na^+^ half saturation constant for *I_NaK_*  Hill coefficient for Na^+^ in *I_NaK_*  K^+^ half saturation constant for *I_NaK_*  Time constant for the Ca^2+^-dependent inactivation  Na/K permeability ratio  Scaling factor for *J_rel_*  Maximum SERCA flux  Half-saturation for forward SERCA  Half-saturation for reverse SERCA  Hill coefficient for forward SERCA  Hill coefficient for reverse SERCA  SR leak rate constant  Time constant for *J_tr_*  Total troponin concentration  Half-saturation for troponin  Total calmodulin concentration  Half-saturation for calmodulin  Total calsequestrin concentration  Half-saturation for calsequestrin  Rate constant for RyR opening  Rate constant for RyR closing  Exponent for RyR gate | 0.76 cm  96.5 C/mmol  8.314 J/mol-K  296 K  1.0 µF/cm^2^  1.8  5.7934 µm  6.0 µm  10.5 µm  1.3854 x 10^-5^ cm^2^  0.0903 pL  0.08127 pL  0.00903 pL  3.94 pL  1796 µM  154578 µM  5400 µM  13818.5982638 µM  130953.391484 µM  4.8 x 10^-5^ dm^3^/(F-ms)  5.4 x 10^-3^ mS/µF  0.0008 mS/µF  0.000039 mS/µF  10^-6^ mS/μF  145 mS/μF  0.02 mS/μF  0.021 mS/μF  0.00007 mS/μF  6.804 x 10^-17^ pA/(pF.µM^4^)  10^-16^ pA/(pF.µM^4^)  0.5  3.83916 pA/pF  186000 μM  3.2  1500 μM  10 ms  0.01833  0.01 ms^-1^  0.9996 ms^-1^  0.5 μM  3500.0 μM  2.0  2.0  5 x 10^-6^ ms^-1^  200 ms  35 μM  0.5 μM  50 μM  2.38 μM  24750 μM  800 μM  1  0.16  4 |

**Initial values**

| Parameter | Initial value |
| --- | --- |
| V  m  h  j  d  f  f_Ca_  r  s  s_slow_  y  b  g  u_a_  u_i_  P_o1_  [Ca]_JSR_  [Ca]_NSR_  [Ca]_i_  Xr  Xs_1_  Xs_2_ | -72.3 mV  0.001729  0.624946  0.624946  0.000109  0.999929  1.001951  0.003223  0.999969  0.999969  0.562306  0.000708  0.914717  0.000554  1.000000  0.002247  790.502388 μM  794.054383 μM  0.137822 μM  0.025742210977  0.012668791315  0.028399873909 |

**Numerical accuracy of CV (cm/s) for different Δx and Δt**

CV was measured at varying space and time steps in simulation domains of the same physical size, but different resolution. Based on these measurements (see table below), we chose Δx=0.00625 cm and Δt=0.02 ms for our 2D simulations as optimal space and time steps. The experimental value of CV was estimated at ~20-25 cm/s.

| Δt, ms | Δx = 0.003125 cm | Δx = 0.00625 cm | Δx = 0.0125 cm |
| --- | --- | --- | --- |
| 0.0025  0.005  0.01  0.02 | 25.3 cm/s  25.0 cm/s  25.0 cm/s  25.0 cm/s | 23.3 cm/s  23.1 cm/s  23.1 cm/s  22.2 cm/s | 18.9 cm/s  18.8 cm/s  18.6 cm/s  18.5 cm/s |

**Reference**

1. Starý T, Biktashev VN (2015) Exponential integrators for a Markov chain model of the fast sodium channel of cardiomyocytes. IEEE Trans Biomed Eng 62(4):1070-6.
