# Supplementary figures and images for: A Mathematical Model of Neonatal Rat Atrial Monolayers with Constitutively Active Acetylcholine-Mediated K+ Current
Source: PLoS Comput Biol. 2016 Jun 22;12(6):e1004946. doi: 10.1371/journal.pcbi.1004946 (PMC4917258; doi:10.1371/journal.pcbi.1004946)

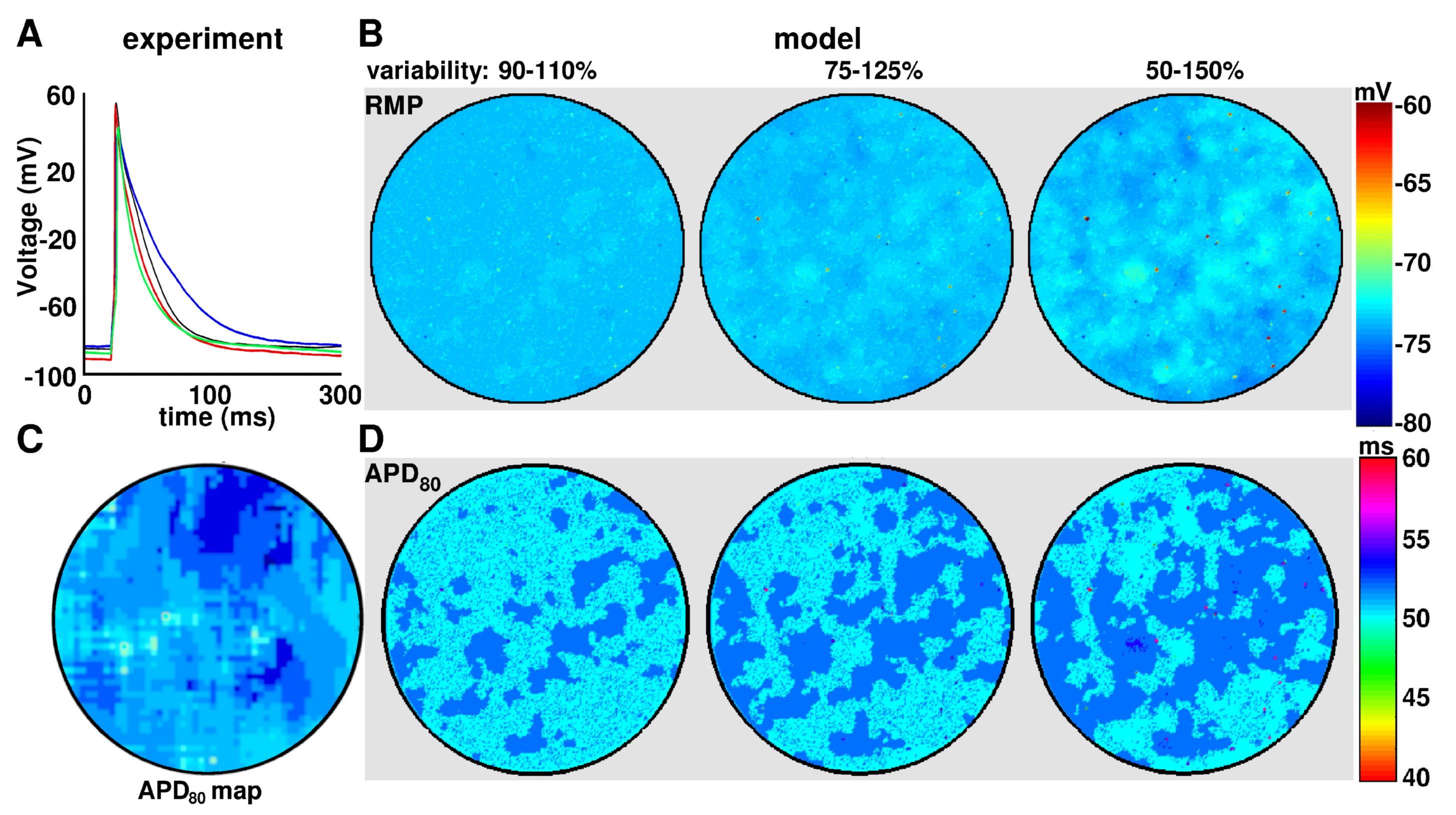

Supplement: S1 Fig — A, Intercellular variability in the patch clamp recordings of 4 APs recorded from 4 different neonatal rat atrial cardiomyocytes, cultured under the same normal growth conditions, in the control environment. B, RMP maps obtained from simulated monolayers with varying levels of intercellular variability. Mean RMP is ~-73 mV in each case with progressively increased dispersion, as range of variability becomes more widespread (left to right). C, A representative APD80 map from our in vitro control monolayers. D, APD80 maps measured from in silico monolayers, corresponding to the RMP maps presented in the upper panel. (TIFF) [file pcbi.1004946.s002.tiff]
